# LATS kinases and SLUG regulate the transition to advanced stage in aggressive oral cancer cells

**DOI:** 10.1038/s41598-022-16667-5

**Published:** 2022-07-20

**Authors:** Emi Fujibayashi, Satomi Mukai, Kosuke Torigata, Yumi Ando, Toshihiro Uchihashi, Masami Nozaki, Susumu Tanaka, Masato Okada, Mikihiko Kogo, Hiroshi Nojima, Norikazu Yabuta

**Affiliations:** 1 Department of Molecular Genetics, Research Institute for Microbial Diseases, Osaka University, Suita, Osaka Japan; 2 First Department of Oral and Maxillofacial Surgery, Graduate School of Dentistry, Osaka University, Suita, Osaka Japan; 3 Department of Cell Biology, Research Institute for Microbial Diseases, Osaka University, Suita, Osaka Japan; 4 Division of Cancer Biology, Aichi Cancer Center Research Institute, 1-1 Kanokoden, Chikusa-ku, Nagoya City, Aichi 464-8681 Japan; 5 Department of Oncogene Research, Research Institute for Microbial Diseases, Osaka University, Yamadaoka 3-1, Suita, Osaka 565-0871 Japan; 6 Present Address: Second Department of Oral and Mexilllofacial Surgery, Osaka Dental University, Hirakata, Osaka 573-1121 Japan

**Keywords:** Oral cancer, Cell invasion, Epithelial-mesenchymal transition, HIPPO signalling, Phosphorylation, Cell growth, Cell migration

## Abstract

The epithelial-to-mesenchymal transition (EMT) is a critical process by which cancer cells acquire malignant features. However, the molecular mechanism and functional implications of EMT and the mesenchymal-to-epithelial transition (MET) in tumor progression remain elusive. In this study, we established two aggressive cancer cell lines from the human oral cancer cell line SAS, mesenchymal-like SAS-m4 and epithelial-like SAS-δ. SAS-δ is a revertant cell obtained by inducing MET in SAS-m4. SAS-δ, but not SAS-m4, exhibited abnormal cell growth, including piled-up overgrowth and invasive tumor formation in the tongues of nude mice, suggesting that SAS-δ represented more advanced cancer cells than the parental SAS cells. EMT-related transcriptional factor SLUG is phosphorylated at T208 and partly stabilized by the Hippo pathway kinases, LATS1 and LATS2. Depletion of SLUG promoted the invasive activity of SAS-δ by increasing the protein levels of LATS1/2 and the proportion of the phosphorylated form among total SLUG protein. Our results suggest that the LATS1/2–SLUG axis regulates the transition of SAS cells to the advanced stage via repeated switching between EMT and MET. Therefore, an anti-SLUG-pT208 antibody would be valuable not alone as a malignant tumor marker antibody but also as a prognostic tool for patients with malignant disease.

## Introduction

The epithelial-to-mesenchymal transition (EMT) is an essential and normal cellular program that converts epithelial cells to mesenchymal-like cells by altering expression of cell adhesion proteins and reorganizing cytoskeletal actin fibers^1^. The apparent reverse of this process, the mesenchymal-to-epithelial transition (MET), is associated with re-epithelialization, which is involved in stem cell reprogramming and secondary (metastatic) tumor formation in distant sites^2^. Notably, however, MET is defined as an independent process, and is intrinsically different from reverse EMT. In fact, it seems that EMT is partially executed by a continuous transition between epithelial and mesenchymal states, and this may confer the plasticity associated with malignant cancer cells^3^. EMT is stringently regulated by EMT transcription factors (EMT-TFs), including SNAIL (*SNAI1*), SLUG (*SNAI2*), and ZEB1/2, through coordination between repression of epithelial genes such as E-cadherin and induction of mesenchymal genes such as vimentin^4,5^. The transcriptional and post-translational regulations of EMT-TFs are dependent on cell type and cellular context^6–8^. However, the molecular details of the correlation between EMT and MET remain unclear.

The Hippo pathway is a pivotal phosphorylation cascade that regulates tissue homeostasis and tumorigenesis by controlling cell proliferation and death in response to a diverse range of stimuli, including cell–cell contact, cell polarity, and mechanical features^9,10^. Upon exposure of mammalian cells to such stimuli, the two core kinases, LATS1 and LATS2, are phosphorylated and activated by upstream kinases, such as MST1, MST2, and MAP4Ks. This in turn promotes the phosphorylation of two transcriptional co-factors, YAP and TAZ, inhibiting their nuclear localization and stabilization and preventing their activation as oncogenes. The Hippo pathway is dysregulated in many human cancers, including oral cancers^11–14^ and involved in cancer progression, especially in the EMT and metastasis^15^. Consistent with this, YAP/TAZ influence the expression patterns of EMT markers as well as cell morphology^16,17^. However, the molecular mechanism of EMT/MET regulation by LATS1/2 remains poorly understood, although it is clear that LATS2 directly phosphorylates SNAIL^18^ and interacts with SnoN, a proto-oncoprotein, to stabilize TAZ during EMT^19^.

## Results

### Higher motility SAS-m4 cells are competent for induction of EMT by TGF-β1

In human oral squamous cell carcinoma (OSCC), including SAS cells, TGF-β1 promotes the motility and invasiveness of these cell lines^20^. We established four SAS-derived cell lines with higher motility, named SAS-m1, SAS-m2, SAS-m3, and SAS-m4, by repeating the motility selection step with TGF-β1 (Fig. S1A). Wound healing assays with TGF-β1 revealed that SAS-m3 and SAS-m4 had acquired higher motility than the parental line (hereafter called p-SAS or SAS) in response to TGF-β1 (Fig. S1B,C).

**Figure 1:**
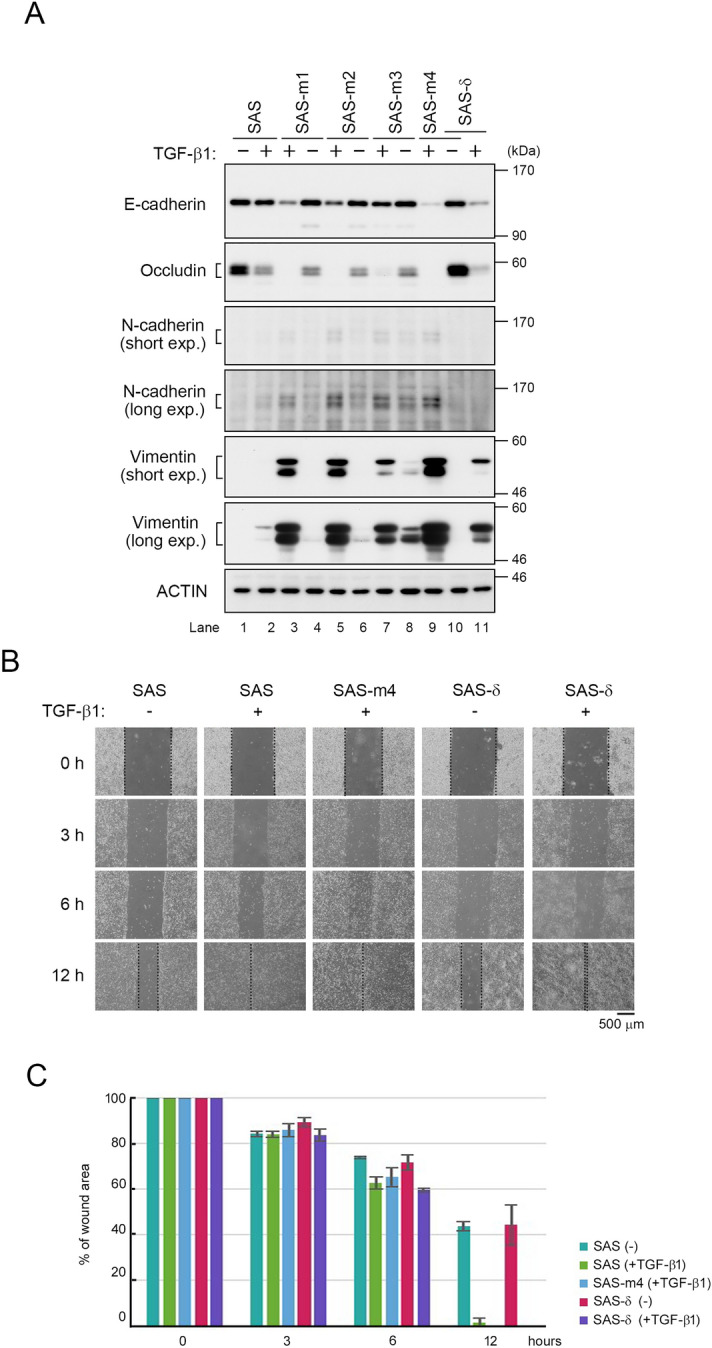
EMT in SAS-m4 cells is highly sensitive to TGF-β1 and partially reversible. (**A**) WB of parental SAS and SAS derivatives with the indicated antibodies against EMT-marker proteins and actin (used as a loading control). SAS-m1, SAS-m2, SAS-m3, and SAS-m4 cell lines were continuously maintained in growth medium containing TGF-β1 (10 ng/ml), and those were cultured in DMEM + 10% fetal bovine serum (FBS) without (–)TGF-β1 for 48 h. Parental SAS and SAS-δ were also treated with (+)TGF-β1 for 48 h (lanes 2 and 11). (**B**) Wound healing assays of parental SAS, SAS-m4, and SAS-δ in the presence (+) or absence (–) of TGF-β1 (10 ng/ml). Images were acquired 0, 3, 6, and 12 h after wounding. (**C**) Quantification of the wound healing assay was performed by measuring the distance across the wound as in Fig. S1C.

We performed western blot analysis (WB) of p-SAS cells and their derivatives SAS-m1, -m2, -m3, and -m4, in the presence or absence of TGF-β1 (Figs. 1A, S2). In the presence of TGF-β1, the expression levels of epithelial markers such as E-cadherin (an adhesion protein) and occludin (a tight junction protein) were markedly lower in the SAS derivatives than those in p-SAS cells, whereas the expression levels of mesenchymal markers such as N-cadherin (an adhesion protein) and vimentin (a cytoskeletal protein) levels were dramatically higher. Notably, the E-cadherin level in p-SAS and SAS-m3 cells were hardly affected by TGF-β1. Moreover, the increase in the level of vimentin was highest in SAS-m4, whereas it was lowest in SAS-m3. Taken together, these results suggested that all of the SAS derivatives were competent for induction of EMT by TGF-β1; thus, we concluded that SAS-m4 was the most suitable derivative for further study.

### Expression of representative EMT markers and cell motility are reversibly altered in SAS-m4 and its revertant, SAS-δ

Although EMT is a potentially reversible program, the molecular mechanism of MET is not thought to represent the strict reversal of EMT^2^. To characterize the differences in molecular mechanisms between EMT and MET, we investigate whether EMT induced in SAS-m4 could be reversed by depletion of TGF-β1. To this end, we cultured and passaged SAS-m4 twice without TGF-β1, yielding a revertant that we named SAS-δ based on its isolation by fourth selection step (Fig. S1A-(x)). SAS-m1, SAS-m2, and SAS-m3 were also cultured in DMEM + 10% FBS without TGF-β1 for 48 h after washing with PBS(−) (Fig. 1A). We then compared the expression levels of representative EMT markers between SAS-δ (without TGF-β1) and SAS-m4 (with TGF-β1). SAS-δ exhibited marked restoration of E-cadherin and occludin expression and a sharp decrease in N-cadherin and vimentin levels (Fig. 1A, lanes 9 and 10). Moreover, when SAS-δ cells were again treated with TGF-β1, the expression levels of E-cadherin and occludin decreased, whereas the levels of N-cadherin did not increase to the level observed in p-SAS treated with TGF-β1 (Fig. 1A, lane 11). Notably, the level of vimentin was higher in TGF-β1-treated SAS-δ than in TGF-β1-treated p-SAS. These results suggest that the changes in expression of EMT markers in SAS-m4 and SAS-δ are partially, but not completely, reversible in response to TGF-β1. Furthermore, wound healing assays revealed that the motility of SAS-δ was promoted by the addition of TGF-β1, similar to the behavior of p-SAS and SAS-m4 with TGF-β1 (Fig. 1B,C); however, a small fraction of SAS-m4 cells moved irregularly and moved into the gap 6 h after the monolayer was scratched (Fig. 1B). These results suggest that the change in motility of SAS-δ is reversible depending on TGF-β1, and that SAS-δ partly recovered an epithelial property, which was tentatively defined based on gene expression pattern^21^, of the parental line without TGF-β1.

### Invasion and migration are suppressed in SAS-δ without TGF-β1

**Figure 2:**
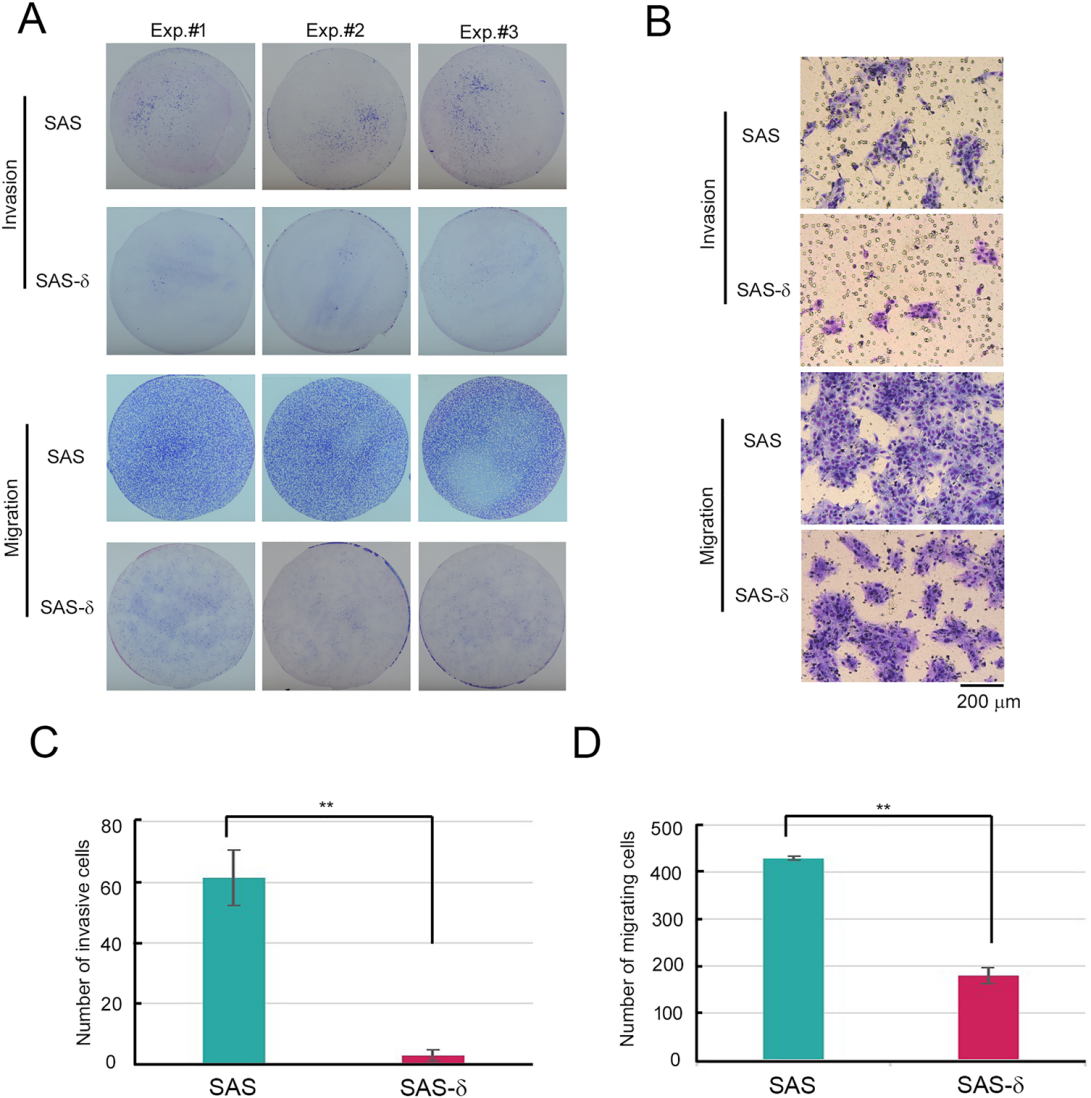
Invasion and migration of SAS-δ are suppressed in the absence of TGF-β1. (**A**) Invasion assays and migration assays of parental SAS and SAS-δ were performed in the absence of TGF-β1. Invading or migrating cells on the membrane were stained, and then the membranes were photographed using a digital camera. (**B**) High-power fields of invading or migrating cells on the membrane were photographed through a microscope. (**C**) and (**D**) Invading (**C**) or migrating (**D**) cells on the membrane were counted on images of the membrane captured through the microscope. Bar graphs show the average number of cells per microscopic field over ten fields (invading cells) or five fields (migrating cells). **P < 0.01.

Next, we examined the invasive and migrating activities of SAS-δ without TGF-β1 using Matrigel invasion chamber (Fig. 2A). The invasion assays revealed that the number of invasive SAS-δ cells was lower than the number of invasive p-SAS (Fig. 2B,C). Moreover, in the migration assays, markedly fewer SAS-δ cells migrated than p-SAS cells (Fig. 2B,D). In the presence of TGF-β1, there was little difference in invasive activity between SAS-δ and p-SAS cells, although the migration of SAS-δ was slightly lower than that of p-SAS (Fig. S3A,B). These results suggest that invasion and migration activities are impaired in SAS-δ without TGF-β1.

### SAS-δ cells pile up and overgrow without cell–cell contact inhibition

**Figure 3:**
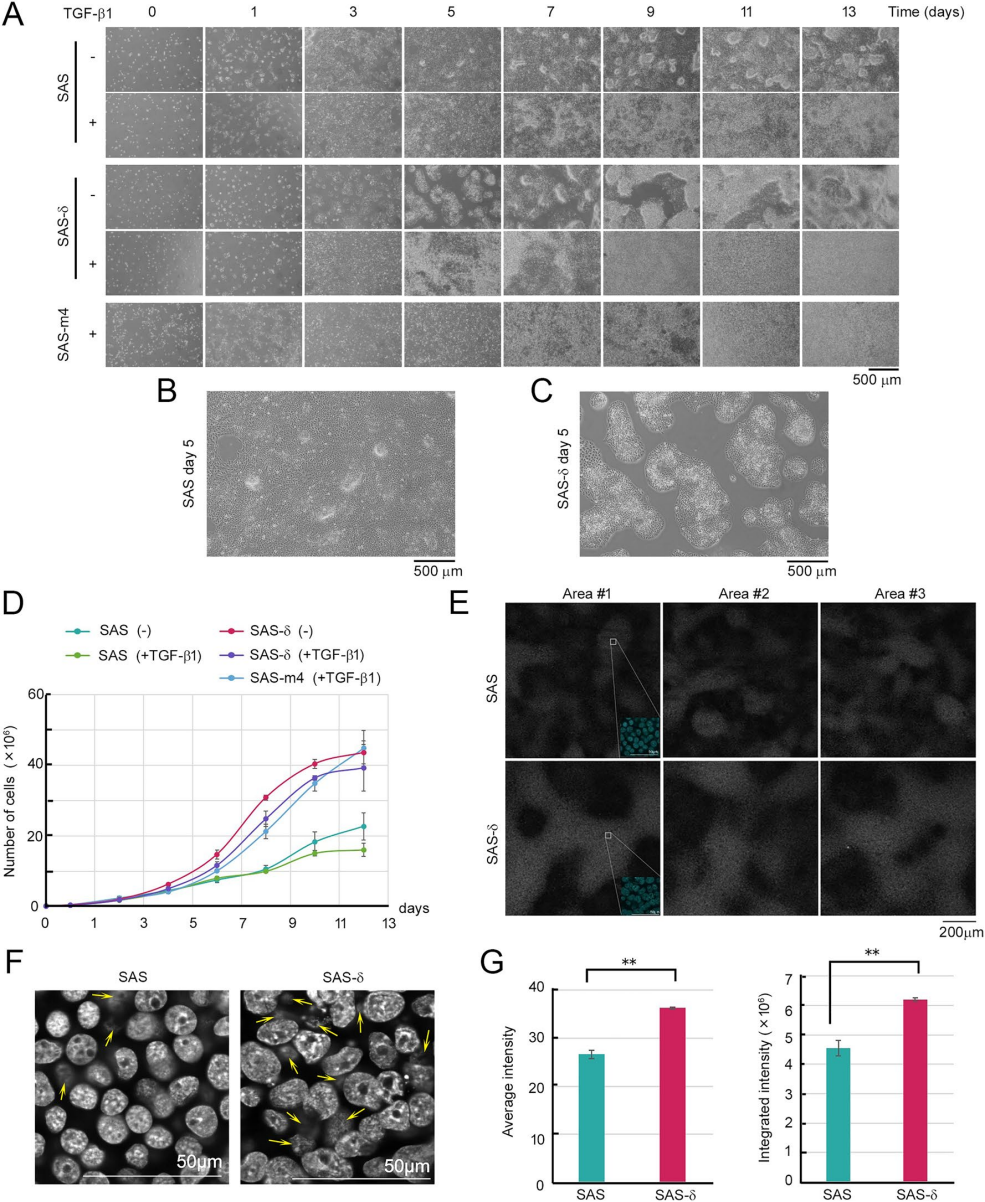
SAS-δ cells pile up and overgrow without cell–cell contact inhibition. (**A**) Parental SAS, SAS-δ, and SAS-m4 were treated with (+) or without (–)TGF-β1 (10 ng/ml) for the indicated numbers of days. Images were captured using a microscope. (**B**) and (**C**) The photograph digitally enlarged images of parental SAS (**B**) and SAS-δ (**C**) on day 5 in the absence of TGF-β1. (**D**) Growth curves of parental SAS, SAS-δ, and SAS-m4 in the presence (+) or absence (–) of TGF-β1. Cells were maintained as in (**A**). Data represent the average of cells from three independent experiments. (**E**) Visualization of the three-dimensional cell density of parental SAS and SAS-δ. A two-dimensional image was reconstructed from three dimensional stacks by superimposing the collected Z-series images of the stained nuclei (pale gray patches). Insets show enlarged color images of nuclei (blue). (**F**) The photograph digitally enlarged monochrome images of nuclei in (**E**). The photo images were focused on nuclei of cells in the upper layer. Yellow arrows indicate out-of-focus nuclei of cells grown in the lower layer, suggesting that the upper cells continuously grew and piled up. (**G**) Bar graphs show the average intensity (left) and integrated intensity (right) of nuclear signals in the reconstructed images (as in **E**) of parental SAS and SAS-δ. Data represent the mean ± standard deviation from three independent areas. **P < 0.01.

To characterize cell growth control in SAS-δ, we first cultured p-SAS and SAS-δ in medium with or without TGF-β1, and then acquired micrographs every 2 days for 13 days (Fig. 3A). In the absence of TGF-β1, SAS-δ continued to grow faster and pile up without spreading in the horizontal direction, whereas p-SAS grew constantly until reaching confluence (Fig. 3B,C). Indeed, the growth curves of these cell lines revealed that SAS-δ cell number increased markedly for 10 days, whereas the number of p-SAS cells increased slowly for 8 days and accelerated slightly thereafter (Fig. 3D). These results suggested that SAS-δ had lost the ability to grow in a spreading manner in a horizontal direction on the bottom surface of standard culture dishes and became unable to induce growth arrest mediated by cell–cell contact. On the other hand, in the presence of TGF-β1, SAS-δ grew widely and as efficiently as the p-SAS cells, spreading until it reached confluence on day 3 or 5 and forming apparent foci after day 5 (Fig. 3A, fourth panels from top). Consistent with this, in the presence of TGF-β1 the growth rates of SAS-δ and p-SAS cells were slightly suppressed, whereas, in the presence of TGF-β1, the numbers of SAS-m4 and SAS-δ continued to increase similarly for 10 days (Fig. 3D).

Next, we grew p-SAS and SAS-δ cells until they piled up (at day 14), stained their nuclei with Hoechst 33258, and acquired images by confocal microscopy (Fig. 3E, pale gray patches; and F). We then quantified staining intensity per unit area in 2D images reconstructed from 3D stacks using image analysis software. The signal intensity of the stained nuclei of SAS-δ cells grown in multilayers is expected to be higher than that of p-SAS cells grown in a near monolayer. Indeed, the signal intensity of piled-up SAS-δ was higher than that of p-SAS cells, which was spread continuously throughout the area (Fig. 3E,G). Taken together, these results suggest that SAS-δ acquired unique properties, such as lower migration/invasion and higher growth rates than p-SAS cells, which could contribute to their biological features, including piled-up overgrowth.

### The Hippo pathway is downregulated in SAS-δ

**Figure 4:**
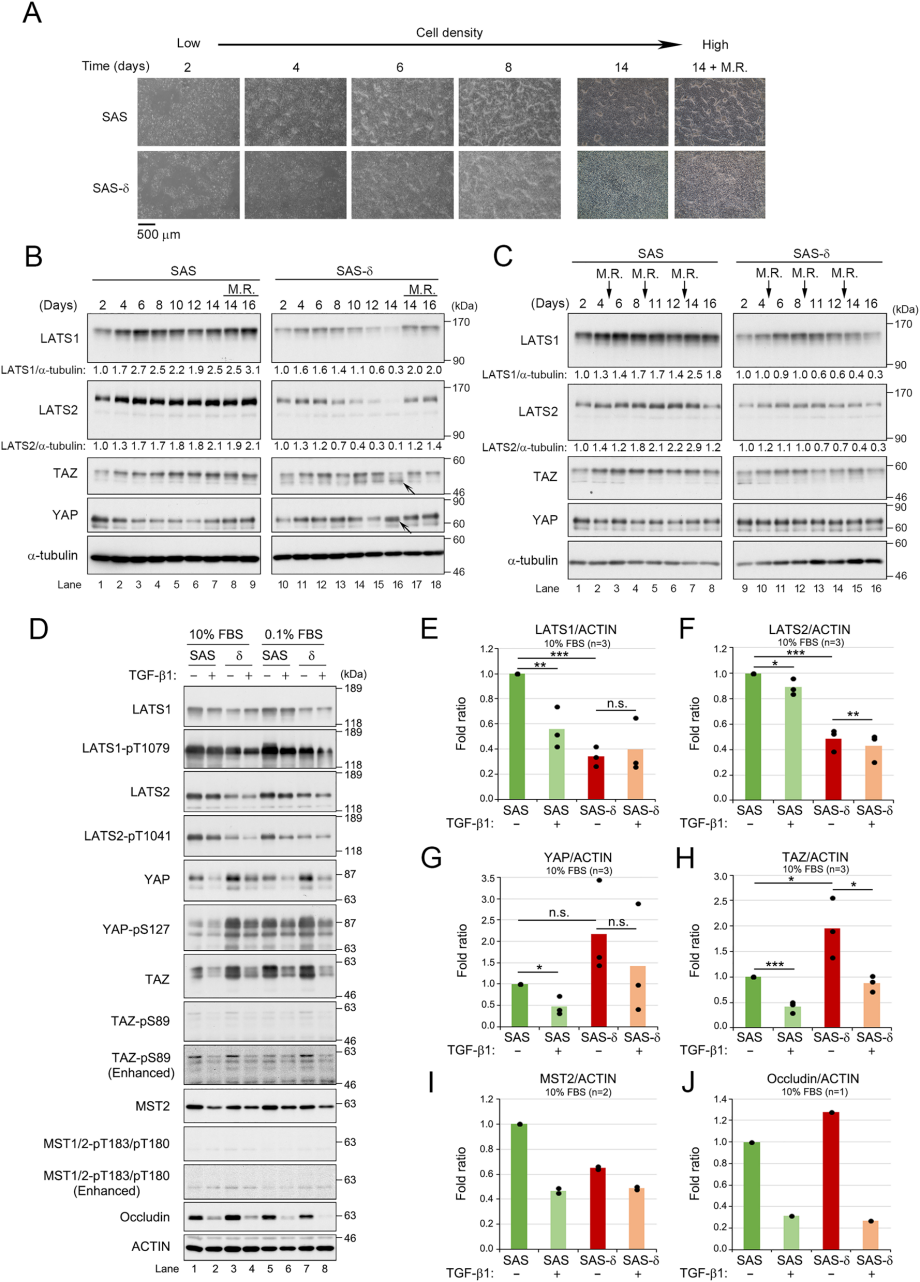
The Hippo pathway is downregulated in SAS-δ. (**A**) Parental SAS and SAS-δ cells (2.0 × 10^6^ cells/dish) were seeded in 60 mm dishes and grown in the absence of TGF-β1 for the indicated number of days. The culture medium of both cell lines was replaced with or without a fresh medium at Day 12, and then cultured for 2 days (Day 14 or Day 14 + M.R.). Images show the morphology of the growing cells. Both cell lines were fully confluent at Day 4, but SAS-δ cells grew continuously and piled up after reaching confluence. M.R., medium replacement. (**B**) and (**C**) WB of parental SAS and SAS-δ, which were grown in the absence of TGF-β1 for the indicated days, as in (**A**). The relative levels of LATS1/2 normalized to the corresponding band intensity of α-tubulin are shown below panels. The normalized band intensities of LATS1/2 at 2 days were defined as 1.0. Original images of duplicate blots were shown in Fig. S8. (**B**) The cultured medium of both cell lines was replaced with or without a fresh medium at Day 12, and then cultured for 2 or 4 days (Day 14, Day 14 + M.R., or Day 16 + M.R.). (**C**) The culture medium of both cell lines was sequentially replaced with fresh medium every 4 days at the time points indicated by arrows (at Day 5, 9 and 13), and then continuously cultured until Day 16. Hippo pathway-related proteins were detected using the indicated antibodies. α-tubulin was used as a loading control. (**D**) WB of parental SAS and SAS-δ cultured with the medium containing 10% or 0.1% FBS in the presence (+) or absence (–) of TGF-β1 (10 ng/ml, 48 h). Hippo pathway-related proteins and phosphorylation levels were detected using the indicated antibodies. Occludin is a TGF-β1-induced EMT marker. Actin was used as a loading control. (**E**–**J**) Average relative levels of the indicated proteins, normalized to the corresponding band intensity of actin are shown in bar graphs (n = 2 or 3), and the respective values are shown in dot plots. n.s., no significant difference.

The canonical Hippo pathway plays a pivotal role in contact-mediated inhibition in normal cells^9^. Duplicate WB revealed that in p-SAS, the expression of not only LATS1/2 but also TAZ was gradually upregulated in response to a higher cell density, and their levels reached a plateau on day 6 or day 8 and remained stable thereafter (Figs. 4A, B, and S8). Conversely, YAP expression was gradually downregulated in response to higher cell density. In SAS-δ, LATS1/2 expression was slightly and gradually upregulated for 6 and 4 days, respectively, but started to decrease on day 8 and day 6, respectively (Fig. 4B, right panels). LATS1/2 were hardly detectable on day 14, but faster migrating bands representing active forms (non- or hypo-phosphorylated forms) of YAP and TAZ were subtle and apparent changes, respectively (Fig. 4B, arrows). Total protein levels of YAP/TAZ did not gradually increase or decrease, but rather slightly fluctuated, during prolonged culture of SAS-δ. Thus, during prolonged culture of SAS-δ, but not of p-SAS cells, LATS1/2 protein levels decreased, and this was accompanied by activation of YAP/TAZ. Moreover, the observation that the protein levels of LATS1/2 and the phosphorylated forms of YAP/TAZ (i.e., the slower migrating bands) increased gradually until day 6 after commencing culture of sub-confluent SAS-δ cells suggests that the canonical Hippo pathway in SAS-δ can respond to cell density unless the levels of LATS1/2 are substantially reduced. Importantly, the decreased intensity bands of LATS1/2 and the faster migrating bands of YAP/TAZ were restored to higher intensity bands and slower migrating bands, respectively, by culturing for 2 days after changing to fresh culture medium on day 12 (Fig. 4B, lane 17). Indeed, the downregulation of LATS1/2 in SAS-δ during prolonged culture was partially rescued by replacement of the culture medium with fresh medium every 4 days (Fig. 4C, right panels). Taken together, these results suggested that the protein levels of LATS1/2 in SAS-δ were downregulated by some extracellular factor(s) in the culture medium, rather than by cell density.

Next, we investigated whether the levels of LATS1/2 and their activated states (pT1079 and pT1041, respectively) in p-SAS and SAS-δ were altered by the presence of TGF-β1. In p-SAS, the levels of LATS1/2, as well as the levels of pT1079/pT1041, were decreased by treatment with TGF-β1 (Fig. 4D–F, n = 3). Moreover, the protein and phosphorylation levels of LATS1/2 in SAS-δ were significantly lower than those in p-SAS with or without TGF-β1 (Figs. 4D–F, S3C,D). The phosphorylations of LATS1-T1079 and LATS 2-T1041 are regulated by MST1/2 kinases^22^. The amount of MST2 protein was lower in TGF-β1-untreated SAS-δ and TGF-β1-treated p-SAS and SAS-δ than in TGF-β1-untreated p-SAS (Fig. 4D, lanes 1–4; 4I, n = 2), although the activating phosphorylation of MST2 (MST2-pT180) did not change significantly in p-SAS and SAS-δ upon addition of TGF-β1.

Furthermore, the levels of TAZ and Occludin proteins were markedly higher in SAS-δ than those in p-SAS (there was no significant increase in YAP level), but their levels decreased upon addition of TGF-β1 (Fig. 4D, lanes 1–4; G,H,J, n = 3 or 1), consistent with a previous report on EMT in mammary gland and breast cancer cell lines^23^. However, the inhibitory phosphorylation level of YAP-S127 normalized to total YAP protein were markedly higher in SAS-δ and TGF-β1-treated p-SAS, which is consistent with the behavior of LATS1-pT1079 in SAS-δ in a Hippo pathway-dependent manner at least under TGF-β1-untreated conditions (Figs. 4D, lanes 1–4, S3C,E). On the other hand, the inhibitory phosphorylation level of TAZ-S89 normalized to total TAZ protein was lower in SAS-δ than in SAS (Figs. 4D, lanes 1–4, S3F). Considering that LATS2-pT1041 was disturbed in SAS-δ (Figs. 4D, S3D), these results suggest that the inhibitory phosphorylation of TAZ by LATS2 is disturbed in SAS-δ and that this leads to aberrant accumulation of TAZ protein; however, it is known that TAZ-pS89 and YAP-pS127 are involved in the cytoplasmic sequestering of these proteins^22^. These results suggest that, in the absence of TGF-β1, TAZ accumulate and become activated due to the decreased levels of LATS1/2 in SAS-δ.

The results shown in Fig. 4B and C suggest the possibility that exhaustion of FBS in the culture medium caused the downregulation of LATS1/2 in SAS-δ. To address this concern, we examined the protein and activation levels of LATS1/2 and other Hippo components in SAS and SAS-δ cells cultured under low serum conditions (0.1% FBS). The levels of LATS1/2, YAP/TAZ, and MST2 were not appreciably affected by low serum conditions (Fig. 4D, lanes 5–8), although the levels of YAP-pS127 were increased in SAS, but not in SAS-δ, with or without TGF-β1. These results suggest that the downregulation of LATS1/2 in SAS-δ is promoted by paracrine and/or autocrine signaling via some secretory factor(s), but not by serum starvation, during prolonged culture. Indeed, abnormal growth of SAS-δ cells, such as piled-up growth, was not affected by low serum conditions, because SAS-δ formed piled-up colonies even under low serum conditions without TGF-β1; however, the colonies were smaller due to slow growth under such conditions (Fig. S3G-(vii)). Notably, the piled-up overgrowth of SAS-δ cells ceased after addition of 10 ng/ml TGF-β1 to the culture medium under both 10% and 0.1% FBS conditions (Fig. S3G-(iv), (viii)).

### LATS1/2 phosphorylate SLUG on T208

**Figure 5:**
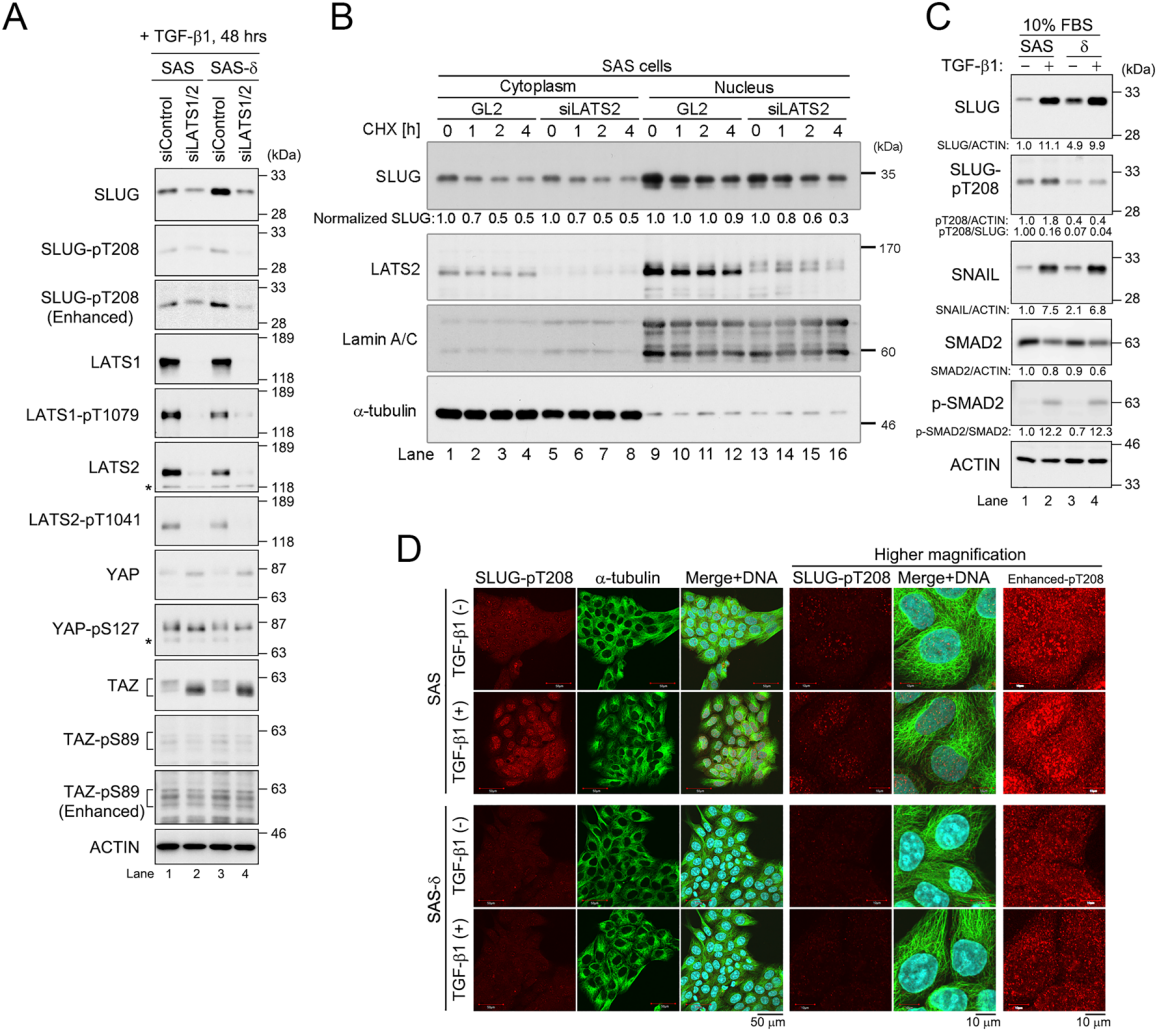
LATS1 and LATS2 phosphorylate SLUG on T208. (**A**) SAS and SAS-δ cells were co-transfected with siRNAs against LATS1 and LATS2 (siLATS1/2), and then cultured with TGF-β1 for 48 h, followed by WB with the indicated antibodies. (**B**) SAS cells transfected with GL2 (as a negative control) or LATS2 siRNA duplex were treated with cycloheximide (CHX) for the indicated periods, and then subjected to subcellular fractionation. Protein levels of SLUG and LATS2 were determined by WB. Lamin A/C and α-tubulin are nuclear and cytoplasmic fraction markers, respectively. The relative levels of cytoplasmic and nuclear SLUG normalized to the corresponding band intensities of α-tubulin and Lamin A/C, respectively, are shown below top panel. The normalized band intensities of SLUG at 0 h after CHX treatment were defined as 1.0 (lanes 1, 5, 9, and 13). (**C**) WB of parental SAS and SAS-δ, which were cultured in medium containing 10% FBS in the presence (+) or absence (–) of TGF-β1 (48 h), was performed with the indicated antibodies. The relative levels of the indicated proteins normalized to the corresponding band intensity of actin, SLUG, or SMAD2, are shown below panels. (**D**) Immunofluorescence staining with anti-SLUG-pT208 (red) and α-tubulin (green) antibodies. Cells were cultured as in (**C**). DNA was visualized by Hoechst 33258 staining (blue). The cell size were comparable between SAS and SAS-δ in the presence or absence of TGF-β1.

SNAIL and SLUG are pivotal regulators of EMT^24^. We found two LATS phosphorylation consensus sequences (H–x–R–x–x–pS/pT) in the zinc-finger motifs of mouse Slug (T183 and T209) and human SLUG (T182 and T208) (Fig. S4A). In vitro kinase assays revealed that LATS2 phosphorylated Slug primarily at T209 (Fig. S4B). To confirm that LATS2 directly phosphorylates T209 of Slug, we performed in vitro LATS1/2 kinase assays and WB with an antibody against phosphorylated T209 (Fig. S4C,D). Because this antibody can also recognize pT208 in human SLUG, hereafter this antibody will be referred to as pT208 antibody. Indeed, the intensity of the band recognized by the pT208 antibody was decreased after knockdown of human SLUG in SAS cells by small interfering RNA (siRNA), and this decrease was prevented by phosphorylated T209 antigen peptides, but not by non-phosphorylated T209 peptides (Fig. S4E). The specificity of the pT208 antibody was also confirmed by siRNA-mediated double knockdown of LATS1/2 in SAS and SAS-δ (Fig. 5A). Together, these results suggest that LATS1/2 phosphorylate SLUG on T208 in vitro and in cultured SAS cells.

The levels of SLUG protein were significantly higher in SAS-δ than in p-SAS, and the higher levels were reduced to below the levels in control scrambled siRNA (siControl) cells by double knockdown of LATS1/2 (Fig. 5A). The levels of SLUG-pT208 were also decreased by LATS1/2 knockdown. Notably, the protein levels of YAP/TAZ were also increased by LATS1/2 knockdown in SAS and SAS-δ, whereas phosphorylation at S127 and S89 was hardly affected by LATS1/2 knockdown. Moreover, cell fractionation and pulse-chase experiments revealed that knockdown of LATS2 decreased the level and stability of nuclear SLUG protein in p-SAS (Fig. 5B, lanes 13–16). Thus, it is likely that LATS1/2 contribute to the stabilization of SLUG protein and the destabilization of YAP/TAZ proteins by the phosphorylation of sites other than S127 and S89 because faster migrating bands of YAP/TAZ proteins were also observed in LATS1/2-knockdown cells. However, the stabilization of SLUG by LATS1/2 may play only a partial role in regulating the levels of total cellular SLUG protein because the level of SLUG in SAS-δ was higher than that in SAS despite the lower levels of LATS1/2 proteins in SAS-δ.

The mRNA and protein levels of SNAIL and SLUG and the activity of SMAD2/3 increase in response to TGF-β1^4,23^. The level of SLUG, as well as SNAIL and phosphorylated SMAD2 (p-SMAD2), increased significantly in p-SAS and SAS-δ cells with TGF-β1 (Fig. 5C, lanes 2 and 4). The levels of SLUG-pT208 were significantly lower in SAS-δ than in p-SAS regardless of the presence of TGF-β1 (Fig. 5C, lanes 3 and 4), although the protein levels of SLUG were higher in SAS-δ and increased by the addition of TGF-β1, whereas p-SMAD2 level was comparable between p-SAS and SAS-δ. Indeed, immunofluorescence staining analyses also revealed that the nuclear accumulation of SLUG protein increased in SAS-δ after the addition of TGF-β1 (Fig. S4F, yellow arrows). On the other hand, SLUG-pT208 signals were localized both to the cytoplasm and the nucleus (in which the signals formed multiple dots) of p-SAS cells (Fig. 5D, top panels). Notably, SLUG-pT208 signals accumulated in the nucleus after addition of TGF-β1 (Fig. 5D, second panels from top), whereas the nuclear signals were significantly lower in SAS-δ cells regardless of TGF-β1 addition (third and fourth panels from top). Taken together, these results suggest that LATS1/2 causes the phosphorylation and accumulation of nuclear SLUG-T208 in p-SAS in a TGF-β1-dependent manner. This is supported by the observation that the level of SLUG-pT208 in SAS-δ expressing low levels of LATS1/2 was lower.

### Loss of SLUG promotes invasiveness of SAS-δ

**Figure 6:**
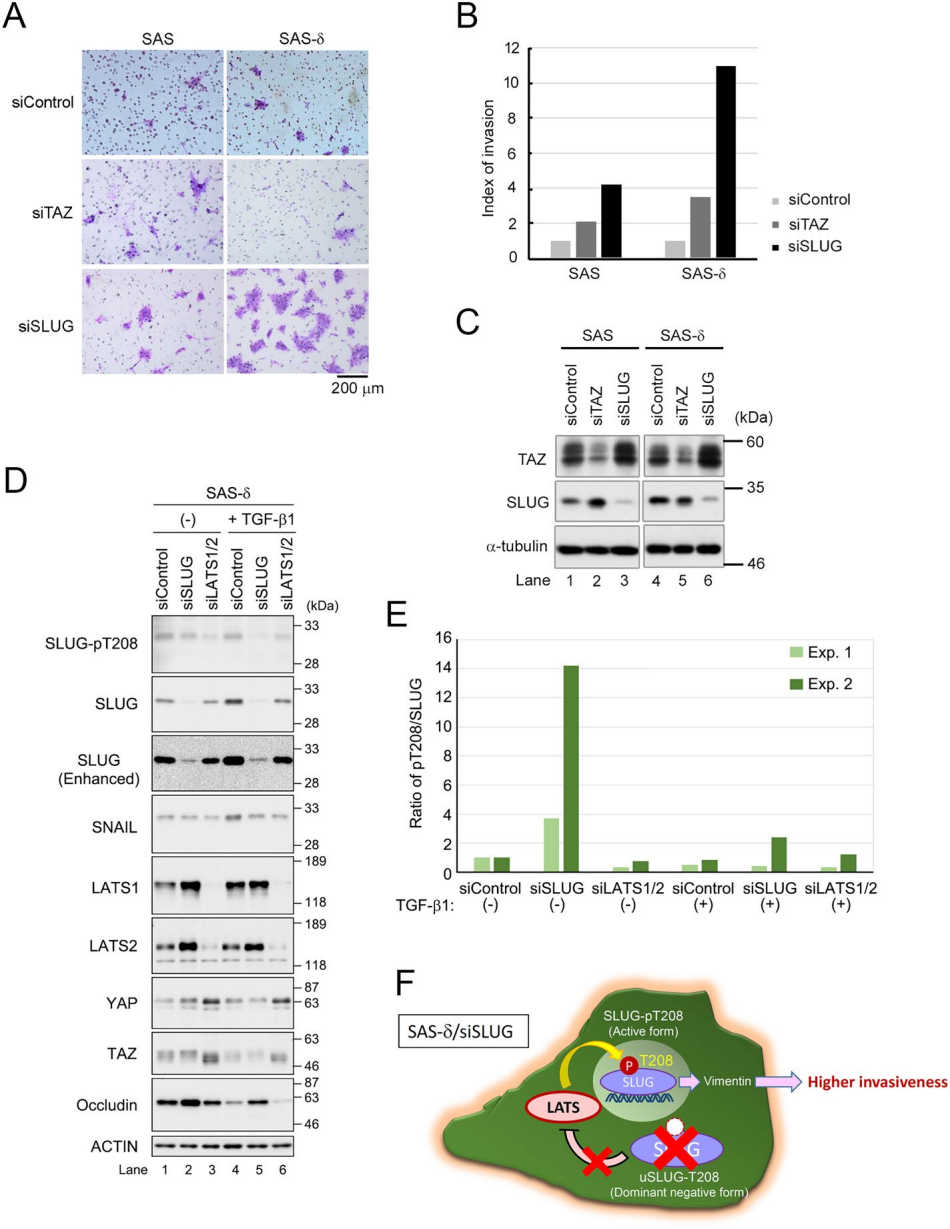
Downregulation of SLUG enhances in vitro invasiveness of SAS-δ via activation of the LATS1/2–SLUG-pT208 axis. (**A**) Invasion assays of parental SAS and SAS-δ transfected with siRNAs against TAZ, SLUG or siControl (negative control) were performed in the absence of TGF-β1 as in Fig. 2A. Invading cells on the membrane were stained, and then the membranes were photographed using a digital camera. (**B**) Bar graphs showing the index of invasion in (**A**), calculated by dividing the percent of invasion (% invasion = the average number of invading cells / the average number of migrating cells × 100) of parental SAS or SAS-δ cells transfected with the indicated siRNAs by the percent invasion of cells transfected with siControl. (**C**) WB of lysates from siRNA-treated cells used in the invasion assays. α-tubulin was used as a loading control. (**D**) SAS-δ cells were transfected with siSLUG, siLATS1/2 or siControl, and then cultured in the presence (+) or absence (–) of TGF-β1 (10 ng/ml) for 48 h, followed by WB with the indicated antibodies. Actin was used as a loading control. (**E**) Bar graph showing the ratio of SLUG-pT208 to total SLUG protein in SAS-δ transfected with siSLUG or siLATS1/2 in the presence (+) or absence (–) of TGF-β1 (10 ng/ml) for 48 h. Two experiments (Exp. 1 and 2, corresponding to those shown in (**D**) and Fig. S6A, respectively) were performed independently. (**F**) A speculated model for the molecular mechanism by which downregulation of SLUG promotes accumulation of T208-phosphorylated SLUG via LATS1/2 activation in SAS-δ. Knockdown of SLUG in SAS-δ increases LATS1/2, thereby efficiently phosphorylating residual SLUG protein on T208 for enhancing the invasiveness of the cells.

To determine whether SLUG or TAZ is involved in invasive activity of p-SAS or SAS-δ, we knocked down SLUG or TAZ with siRNA and performed migration/invasion assays without TGF-β1 (Fig. 6A–C). Invasion by p-SAS was slightly increased upon depletion of TAZ (approximately twofold higher than in the siControl) and was increased further upon depletion of SLUG (approximately fourfold greater than siControl). Invasion by SAS-δ was markedly increased by depletion of SLUG (approximately 11-fold higher than in the siControl), but only slightly increased by the depletion of TAZ (approximately threefold higher than in the siControl) or LATS2 (Fig. S5A,B; approximately twofold higher than in the siControl). On the other hand, invasion by SAS-δ was hardly affected by depletion of SNAIL (Fig. S5A, B). These results suggest that overexpression of SLUG, particularly its T208-unphosphorylated form (hereafter named uSLUG-T208), might suppress the invasive activity of SAS-δ because the depletion of SLUG in SAS-δ, which has lower migration and invasion activity than p-SAS, dramatically increased invasion by SAS-δ.

A decrease in the SLUG level allowed SAS-δ to initiate more invasion than p-SAS (Fig. 6B). However, it is well-known that SLUG induces EMT in epithelial cells and at least partial EMT is crucial for promoting invasion of cancer cells^3^. To resolve this discrepancy, we further examined the molecular relationship between LATS1/2 and SLUG in SAS-δ. WB revealed that the levels of LATS1/2 proteins were increased by knockdown of SLUG in SAS-δ (Fig. 6D, lane 2). Interestingly, the phosphorylation level of T208 on SLUG was maintained even after the successful knockdown of SLUG without TGF-β1 (Figs. 6D, S6A). Indeed, we validated that Slug-pT208 levels normalized to total Slug proteins levels were significantly increased by knockdown of SLUG without TGF-β1 (Fig. 6E). In the two independent experiments, only slight differences in the knockdown efficiencies might be reflected in the quite different pT208/SLUG ratios because the decreased level in the denominator (the knockdown efficiency of SLUG) affects the increase in the numerator (the increased phosphorylation level of SLUG-pT208 via LATS1/2 activation). These results suggest that the ratio of SLUG-pT208 to total SLUG was markedly increased by siRNA-mediated inhibition of de novo SLUG synthesis and LATS1/2-mediated increases in the stability of SLUG-pT208 protein. In the presence of TGF-β1, SLUG knockdown failed to maintain the levels of SLUG-pT208 in SAS-δ probably because the rapid turnover of SLUG was promoted by TGF-β1 (Figs. 6D, lane 5; S6A). We also confirmed that the levels of SLUG-pT208 decreased after double knockdown of LATS1/2 (Figs. 6D, lanes 3 and 6; S6A). Moreover, SNAIL expression was not increased by SLUG knockdown in SAS-δ (Fig. 6D), although SNAIL expression was previously reported to be upregulated after the introduction of SLUG siRNAs into some OSCC cell lines^25^. Taken together, these results indicate that SLUG could potentially downregulate LATS1/2 expression, thereby reducing LATS1/2-mediated SLUG-pT208 and eventually leading to the inhibition of SAS-δ cell invasion through the accumulation of uSLUG-T208 as a dominant negative form in SAS-δ (Fig. 6F). Therefore, a reduction in SLUG expression could lead to increased invasion by SAS-δ via the nuclear accumulation of SLUG-pT208 (probably the active form) and the elimination of uSLUG-T208 (probably the inactive form) because SLUG-pT208, which is more stable than uSLUG-T208, preferentially forms prominent nuclear foci (Fig. 6F).

To demonstrate the capability of SLUG-pT208 in EMT, we examined the expression levels of representative EMT markers, such as E-cadherin, N-cadherin, and vimentin, in SAS-δ expressing SLUG siRNA or the SLUG-T208A mutant (Fig. S6). Unexpectedly, E-cadherin levels were hardly affected by knockdown of SLUG or LATS1/2 in SAS-δ without TGF-β1 (Fig. S6A, lanes 1–3). Since N-cadherin and vimentin were present at extremely low or undetectable levels in p-SAS and SAS-δ under TGF-β1-untreated conditions, we could not assess the impact of SLUG knockdown on the expression of these EMT markers (see also Fig. 1A). However, we found that vimentin expression, but not N-cadherin expression, was significantly decreased by knockdown of SLUG or LATS1/2 in SAS-δ treated with TGF-β1 (Fig. S6A, lanes 5 and 6). Indeed, overexpression of the SLUG-T208A mutant decreased the level of vimentin more efficiently than overexpression of SLUG-wild type (WT) in SAS-δ without TGF-β1 (Fig. S6B, lane 4; C–E), whereas the vimentin expression was not induced by transfection with other nucleic acids (Fig. S6A, lanes 1–3), suggesting that uSLUG-T208 actually inhibits EMT, including invasive activity, via suppression of vimentin expression in SAS-δ. Therefore, uSLUG-T208 might be required for the maintenance of epithelial-like phenotypes, especially in the advanced stages of epithelial cancer (Fig. 6F).

On the other hand, we independently established a new SAS-δ from different batch of p-SAS (named SASII for convenience) in the same manner as SAS-δ establishment to demonstrate the reproducibility of data on SAS-δ (Fig. S6F–I). We successfully established SASII-δ from parental SASII after four sequential screening steps in the presence of TGF-β1 and subsequent removal of TGF-β1. Indeed, SASII-δ cells exhibited a morphology with piled-up overgrowth after removal of TGF-β1 from their precursor cell, SASII-m4, which is similar to a biological feature of SAS-δ (Fig. S6F). Moreover, WB revealed that occludin was decreased in SASII-m1, -m2, -m3, and -m4, whereas vimentin was increased in SASII-m1, -m2, and -m3 in the presence of TGF-β1, which is similar to the case of SAS-m1, -m2, -m3, and -m4 (Fig. S6G). Interestingly, in SASII-m1, -m2, -m3, and -m4, vimentin was expressed with a regular molecular weight size (54 kDa) and a slightly smaller molecular size. The shorter band of vimentin was also observed in SAS-δ, but only the shorter vimentin was markedly expressed in SASII-δ. Notably, like SAS-δ, SASII-δ exhibited lower levels of LATS1/2 proteins than those in p-SAS (Fig. S6G, H). However, in the absence of TGF-β1, the levels of YAP/TAZ proteins were more decreased in SASII-δ than those in p-SAS and SAS-δ, suggesting that LATS1/2–YAP/TAZ axis may be largely dispensable for the piled-up overgrowth of SAS-δ and SASII-δ (Fig. S6H). Furthermore, like SAS-δ, the levels of LATS1/2 proteins were increased by knockdown of SLUG in SASII-δ, and the phosphorylation level of T208 on SLUG was maintained in SASII-δ even after dramatically reduction of SLUG by knockdown in the absence of TGF-β1 (Fig. S6I), although the levels of SLUG in SASII-δ were similar to those in SAS and SAS-δ (Fig. S6H). These results indicate that several properties of SAS-δ could be reproduced in independently established SAS-derived cell line, such as SASII-δ.

### SAS-δ exhibits higher invasive tumor growth than parental SAS in vivo

**Figure 7:**
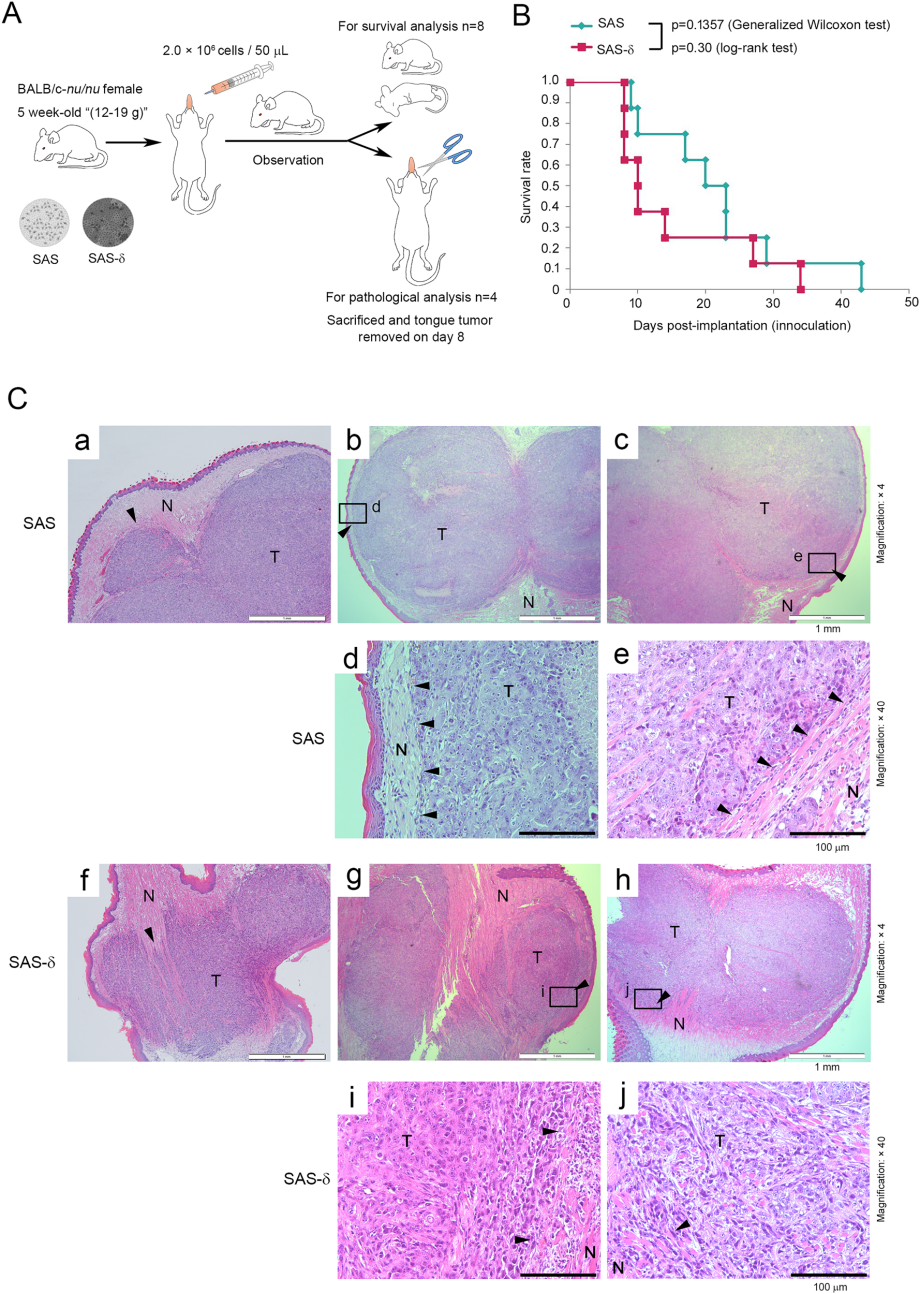
SAS-δ cells enhance in vivo tumorigenicity. (**A**) Schematic drawings of an orthotopic mouse xenograft model of tongue cancer. Parental SAS or SAS-δ cells were injected into the tongue of 5-week-old female nude mice (body weights, 12–19 g; n = 12). These mice were monitored for survival analysis (n = 8) or sacrificed on day 8 for pathological analysis of tongue tumors (n = 4). (**B**) Survival analysis of nude mice injected orthotopically with parental SAS or SAS-δ cells. Survival rates of eight nude mice were evaluated using the Kaplan–Meier method and compared by the generalized Wilcoxon test and log-rank test. (**C**) Representative H&E-stained sections of primary tongue tumors from nude mice injected with parental SAS (a–e) or SAS-δ cells (f–j). Panels d, e, i, and j are high-power fields of areas shown by open boxes in b, c, g, and h, respectively. Arrowheads indicate the boundaries between tumors (T) and normal tissues (N). Scale bars, 1 mm (× 4 magnification).

To determine whether the lower in vitro invasiveness of SAS-δ was reflected in in vivo invasive tumor growth, we injected p-SAS or SAS-δ into the tongues of BALB/c nude mice (Fig. 7A). Mice injected with SAS-δ generated larger tumors on their tongues, and the survival of these mice was markedly reduced, but not significantly different from that of mice injected with p-SAS (Fig. 7B). However, all tumor xenograft samples (day 8, n = 3) containing p-SAS exhibited a distinct boundary between invasion front and normal tongue tissue (transplant site) (Fig. 7C-a–e, arrowheads), whereas all samples (day 8, n = 4) of SAS-δ had an indistinct boundary (Fig. 7C-f–j, arrowheads). In the SAS-δ-containing tissues, there were many areas where the boundaries were hardly recognizable (Fig. 7C-j). Notably, tissue samples containing SAS-δ showed more cells with darkly staining nuclei than samples containing p-SAS, and the abnormal cells were diffusely spread throughout the tissue (Fig. 7C-i and j). These results suggest that compared to the p-SAS cells, SAS-δ exhibits higher invasive tumor growth in vivo, although it is less invasive than p-SAS without TGF-β1 in vitro. This suggests that SAS-δ can drive aggressive malignancy more strongly than p-SAS, and that SAS-δ can generate invasive tumors in vivo in response to various growth factors, including TGF-β1, in the tumor microenvironment and in response to accidental DNA damage that induces genetic mutations or epigenetic abnormalities that suppress SLUG expression (which ultimately allows stable SLUG-pT208 proteins to concentrate efficiently at their transcriptional target sites on chromatin) in oral cancer cells.

## Discussion

Previously, another group established two SAS cell lines with low invasive potential (SAS-L1) and high invasive potential (SAS-H1) by selection based on invasive capacity, a method distinct from the one used in the present study^26^. Nevertheless, SAS-δ exhibited low migration and invasiveness in vitro without TGF-β1 (Fig. 2). Moreover, the abnormal biological properties observed in SAS-δ were not present in SAS-H1 and SAS-L1. Thus, it is likely that SAS-δ is dissimilar to SAS-H1 or SAS-L1, and unique in its capacity for abnormal growth and malignancy. Therefore, it is most likely that SAS-δ are epithelial-like, rather than mesenchymal-like or metastable (i.e., the intermediate stage of EMT); however, the epithelial state of SAS-δ is apparently different from that of p-SAS. These results support a recent model that MET is not simply the reverse of EMT^2^.

On the basis of these results, we propose a new model for the evolution of cancer cells (Fig. S7). Namely, cancer cells change to a more advanced stage by progressively repeating EMT and MET, without metastasis to distant sites, through the following steps: (1) epithelial cancer cells (corresponding to p-SAS) turn into mesenchymal-like cancer cells (corresponding to SAS-m4) via induction of EMT (e.g., addition of TGF-β1); (2) mesenchymal-like cancer cells turn into advanced epithelial (aE) cancer cells (corresponding to SAS-δ) via induction of MET (e.g., depletion of TGF-β1); and (3) aE cancer cells turn into advanced mesenchymal-like (aM) cancer cells via induction of secondary EMT. The grade of malignancy may increase as these steps are repeated. Therefore, aM cancer cells might acquire more aggressive malignant properties than mesenchymal-like cancer cells.

What is the key factor in the molecular machinery involved in switching between EMT and MET in this model of cancer progression? In OSCC cell lines such as Cal27 and HN4, knockdown of TAZ inhibits cell motility, migration, invasion, and the expression of vimentin, whereas conversely the enforced expression of TAZ promotes these features, indicating that TAZ confers EMT-like changes on the cells. TAZ seems to be involved in TGF-β-mediated EMT^27^. Moreover, the promoters of *LATS1/2* genes are methylated in the tumors of OSCC patients at a comparatively early stage or a well-differentiated stage^28^. Furthermore, we previously showed that LATS1/2 are required for spheroid formation of cancer stem-like cells^29^. Taken together with the data that LATS1/2 directly regulate two EMT-TFs, including SLUG during MET or subsequent epithelial stage (Figs. 5, 6) and SNAIL during EMT^18^, it is possible that when the Hippo pathway-mediated growth inhibitory system is disrupted by genetic mutation or oncogenic stress, the core components of this pathway become independent of one another and might play distinct and independent roles in EMT and cancer progression via interactions with new partners^30^. Indeed, YAP and/or TAZ have the ability to interact with SNAIL, SLUG, and/or ZEB1 during development or cancer progression^31,32^, whereas LATS1/2 also regulate chromosomal instability^33,34^.

Interestingly, SLUG and vimentin promote cell migration and invasion through the upregulation of a receptor tyrosine kinase Axl in breast cancer cells^35^, and the overexpression of Axl also correlates with poor prognosis in OSCC patients^36^. Based on our proposed model (Fig. S7), repeated exposure of epithelial cancer cells in oral tumors to TGF-β1 (that is, repeated switching between the EMT and MET mediated by fluctuations in the extracellular TGF-β1 concentration in tumors) might allow these cells to develop into aE cancer cells with potentially higher malignancy, akin to SAS-δ. Importantly, when SLUG expression is accidentally suppressed by gene mutations or epigenetic dysregulation in such aE cancer cells, the proportion of stable SLUG-pT208 may increase markedly, thereby inducing secondary EMT and generating highly invasive mesenchymal-like cancer cells (aM) in the tumor.

In this study, we used a heterogeneous cell population of SAS-δ cells. Since cancer tissue consists of a heterogeneous cell population, we examined the malignant transformation of the entire cancer tissues including heterogeneous cell population considering a clinical-like environment. Moreover, we found an exogenous factor (unknown autocrine soluble factor) that reduces the protein level of LATS2 in heterogeneous SAS-δ cells, and in the future we aim to establish that this is associated with an increased risk of malignancy for the heterogeneous population of cancer cells as a cancer microenvironment using this experimental system with SAS-δ.

## Materials and methods

### Cell culture

SAS cells, a human OSCC cell line derived from a tongue tumor^29^, were obtained from the Human Science Resource Cell Bank (Osaka, Japan) and RIKEN BRC (Ibaraki, Japan). Cells were cultured with Dulbecco’s modified Eagle’s medium (DMEM, Sigma, St. Louis, MO, USA) supplemented with 10% or 0.1% FBS (Hyclone, Logan, UT, USA), 100 U/ml penicillin, and 100 μg/ml streptomycin, and incubated at 37 °C in 5% CO_2_. To establish an SAS-derived cell line with higher motility (Fig. S1A), parental SAS cells were grown to a confluent monolayer in a standard culture dish in the presence of 10 ng/ml recombinant human TGF-β1 (PEPROTECH, Rocky Hill, NJ, USA) and 10% FBS, and then removed the cells in the inner circle (~ 80% of the area) using a cell scraper. Next, after allowing a proportion of the cells in the peripheral region to move into the vacated space (incubation for approximately 7 days), the residual peripheral cells were scraped off. The cells in the interior were then allowed to grow to a confluent monolayer in medium containing TGF-β1. The cells were then scaled up in a 10 cm dish and cultured for approximately 10 days; these cells, selected based on their motility, were named SAS-m1 (Figs. 1A and S1). By repeating this selection two, three, or four times without freeze and thaw steps, we established SAS-derived cells with higher motility, named SAS-m2, SAS-m3, and SAS-m4, respectively. SAS-δ cells were established by culturing and passaging SAS-m4 twice without TGF-β1. Independently of these cell lines, SASII-δ and SASII-derived cells such as SASII-m1, -m2, -m3, -m4, were established from different batch of parental SAS in the same manner as SAS-δ establishment. All these cells were frozen and stored in a polyclonal state after completion of establishment of the cell lines.

### Induction of EMT/MET and cell–cell contact inhibition in SAS

To induce EMT, SAS cells were treated with DMEM + 10% FBS containing 10 ng/ml TGF-β1. To induce MET, the mesenchymal-like cells were cultured in TGF-β1-free DMEM + 10% FBS for 2 days after twice washing with PBS(−). To determine the effects on contact inhibition, SAS and SAS-δ cells were seeded in 60 mm dishes (2.0 × 10^6^ cells/dish) and cultured at 37 °C in 5% CO_2_ for the indicated days. The day after seeding was defined as day 0. The cells were photographed and collected on day 2, 4, 6, 8 and 14, and then subjected to western blotting. For inhibition of protein synthesis in Fig. 5B, cells were incubated with 50 μg/ml cycloheximide (Nacali Tesque, Kyoto, Japan) for the indicated times.

### Wound healing assay

The cells were maintained at confluence. After washing with PBS(−), the cell surface was uniformly scratched with a 1 ml pipette tip. Floating cells were removed by washing with PBS(−), and then adherent cells were cultured at 37 °C in 5% CO_2_ for the indicated times. Cell motility was assessed by measuring the width (distance between both edges of the wound) at the indicated times. Five points of the wound were selected and measured. The values were averaged and expressed relative to the mean value at 0 h after the addition of TGF-β1 (the wound area at 0 h was defined as 100%). Each value is the mean ± standard error from triplicate experiments.

### Antibodies

Anti-SLUG-pT208 polyclonal antibody was raised against phosphorylated T208 of human SLUG (amino acid sequence IRTH(pT)GEKPFS; this sequence is identical to T209 of mouse Slug). Briefly, rabbits were injected with the KLH-conjugated phosphopeptides, and antisera were affinity-purified using a phospho-antigen peptide column. To eliminate reaction of non-specific antibodies with unphosphorylated antigen peptide, the antibody preparation was passed through a column containing non-phosphorylated peptide (SLUG-T208, IRTHTGEKPFS). Generation and purification of the antibody was supported by GenScript (Piscataway, NJ, USA). The commercial antibodies used in the study are listed in Supplementary Table S1.

### Dot blot assay

Antigen peptides containing phosphorylated T208 site or non-phosphorylated peptides were blotted at the indicated concentrations on a PVDF membrane, followed by probing with anti-SLUG-pT208 antibody.

### Western blot analysis

Preparation of whole cell lysates and Western blotting (WB) were performed as described previously^37^. Chemiluminescent signals were detected by ImageQuant LAS4000mini system (Cytiva, Marlborough, MA, USA) or X-ray films (Fujifilm, Tokyo, Japan). Full scan images of all western blots (original blots), including replicates, were shown in Fig. S8. Most of the blots were cut prior to hybridization with antibodies, and their membrane edges failed to be visible because of high signal-to-noise ratio for hybridization with antibodies by successful pre-blocking with TBST + 5% skim milk at room temperature for 1 h.

### siRNAs

Sequences of siRNA duplexes were as follows: siTAZ, 5′-AGAGGUACUUCCUCAAUCAdTdT; siSLUG, 5′-CCCAUUCUGAUGUAAAGAAAUACdCdA; siSNAIL, 5′-UCAACUGCAAAUACUGCAACAAGdGdA-3′; siLATS1, 5′-ACUUUGCCGAGGACCCGAAdTdT-3′; and siLATS2, 5′-CCGCAAAGGGUACACUCAACUCUGUdTdT-3′; and a negative control siRNA against firefly luciferase (GL2), 5′-CGUACGCGGAAUACUUCGAdTdT-3′. As another negative control, scrambled siRNA (siControl, 5′-CGUUAAUCGCGUAUAAUACGCGUdAdT-3′) was purchased from Origene (Rockville, MD, USA).

### Transfection

The siRNA duplexes were transfected using Lipofectamine 2000 (Invitrogen, San Diego, CA, USA). Plasmid DNAs (pCMV6myc-human SLUG-WT and -T208A) were transfected using Lipofectamine and PLUS reagents (Invitrogen). Cells were cultured at 37 °C in 5% CO_2_ for 48 h after replacing the medium with fresh medium (with or without 10 ng/ml TGF-β1), according to the manufacturer’s instructions.

### Cell invasion and migration assays

Cell invasion assays were performed using a two-layer structure Transwell chamber (Corning BioCoat Matrigel invasion chamber, 8.0 μm pore size; Thermo Fisher Scientific, Waltham, MA, USA). Briefly, the cells were seeded in the upper chamber at a density of 5.0 × 10^5^ cells/ml in 2 ml of DMEM without serum, and DMEM containing 10% FBS with (Fig. S3A, B) or without (Figs. 2 and 6) 10 ng/ml TGF-β1 was added to the lower chamber. After incubation at 37 °C in 5% CO_2_ for 72 h, cells on the upper chamber were removed by scrubbing with a cotton-tipped swab. The cells on the lower surface of the membrane were stained using a Diff-Quik kit (Sysmex, Kobe, Japan). Migration assays were performed using a two-layer Transwell chamber (Falcon cell culture inserts; Thermo Fisher Scientific). The number of invading or migrating cells on the membrane was counted on images of the membrane captured through a microscope (Leica Microsystems GmbH, Germany). The numbers of invading or migrating cells are represented as the average number of cells per microscopic field over ten or five fields, respectively.

### Cell growth assays

Cells were seeded at a density of 0.5 × 10^5^/well in 6-well plates, and cultured at 37 °C in 5% CO_2_. Cells in separate wells for each time point were trypsinized and counted on a Countess automated cell counter (Invitrogen) every 2 days for 2 weeks. Day 0 was defined as the day after seeding.

### Indirect immunofluorescence staining

Cells were cultured on coverslips immersed in medium in a culture dish (35 mm) and fixed at room temperature in a sequence of 4% formaldehyde in PBS(−), 0.1% Triton X-100 in PBS(−), and 0.05% Tween-20 in PBS(−) for 10 min each. After removal of the final fixation solution, the fixed cells were blocked for 1 h at room temperature with 2 ml of TBST buffer supplemented with 5% FBS. Subsequently, the cells were incubated with the indicated primary antibodies, followed by incubation with Alexa Fluor 488- and 594-conjugated anti-rabbit IgG (Molecular Probes, Eugene, OR) in TBST containing 5% FBS. For staining with anti-SLUG-pT208 antibody, cells were fixed at − 20 °C in ice-cold methanol for 10 min after treating with a microtubule stabilizing buffer (80 mM PIPES-KOH [pH 6.8], 5 mM EGTA, 1 mM MgCl_2_, 0.5% Triton X-100) at room temperature for 5 min, and then washed twice with PBS(-). The stained cells were observed on a FV10i confocal laser scanning microscope (Olympus, Tokyo, Japan).

### In vitro kinase assay

Recombinant human LATS1 or LATS2 kinase (Carna Biosciences, Hyogo, Japan) was incubated at 30 °C for 30 min with GST-fused mouse Slug-WT, -T183A, -T209A, and all-A (S101A, S139A, S161A, T183A, S196A, T209A, S215A, S248A, and S252A) mutants in LATS1/2-kinase buffer (20 mM PIPES [pH 6.8], 4 mM MnCl_2_, 1 mM DTT, 1 mM NaF, 1 mM Na_3_VO_4_) containing 20 μM ATP and 10 μCi [γ-^32^P] ATP. Reactions were resolved by SDS-PAGE followed by autoradiography and staining of minigels using SimplyBlue SafeStain (Invitrogen, Carlsbad, CA, USA).

### Tongue tumor model of mice

SAS or SAS-δ cells (2 × 10^6^ cells in 50 µl of serum-free DMEM) were injected into the tongues of 5-week-old female nude mice (BALB/c-nu/nu; Japan SLC Inc., Shizuoka, Japan) using a syringe with a 26 G needle. For pathological examination, four mice were sacrificed and their tongue tumors were excised 8 days after tumor implantation. The sections (3 µm thick) of pathological samples were prepared by The Research Foundation for Microbial Diseases of Osaka University (BIKEN) after fixation with 10% formalin neutral buffer solution (Wako Pure Chemical Industries, Ltd., Osaka, Japan). For hematoxylin and eosin (H&E) staining, the sections of mouse tongue tumor specimens were stained using a Tissue-Tek-Prism automated slide stainer (Sakura Fine Japan Co., Ltd., Tokyo, Japan). In brief, after deparaffinization and rehydration, the samples were soaked in hematoxylin solution (Sakura Fine Japan) for 5 min. After washing with water, the samples were soaked in eosin Y solution (WALDECK GmbH & Co. KG, Münster, Germany) for 5 min.

### Ethical permission

The animal experiments were performed in compliance with the ARRIVE guidelines (https://arriveguidelines.org). All animal experiments were approved by the Animal Care and Use Committee of the Research Institute for Microbial Diseases, Osaka University, Japan (approval code: #Biken-AP-H24-17-0 and #Biken-AP-H29-12-0). The methods were carried out in accordance with the approved guidelines.

### Statistical analysis

Statistical analysis was performed in Microsoft Excel. Error bars for data on cell growth and invasion/migration assays represent standard deviation from the mean. *P*-values were calculated using Student’s *t*-test. Survival analysis in vivo was performed with Kaplan–Meier curves, and the comparisons were evaluated by generalized Wilcoxon test and log-rank test. *P < 0.05; **P < 0.01; ***P < 0.001.

## Supplementary Information

Supplementary Information.

## Data Availability

All data generated or analyzed during this study are included in this published article and its supplementary information files.
