# Supplementary material for: LATS kinases and SLUG regulate the transition to advanced stage in aggressive oral cancer cells
Source: Sci Rep. 2022 Jul 20;12:12363. doi: 10.1038/s41598-022-16667-5 (PMC9300623; doi:10.1038/s41598-022-16667-5)
Supplement: Supplementary file 1 — Supplementary Information. [file 41598_2022_16667_MOESM1_ESM.pdf]

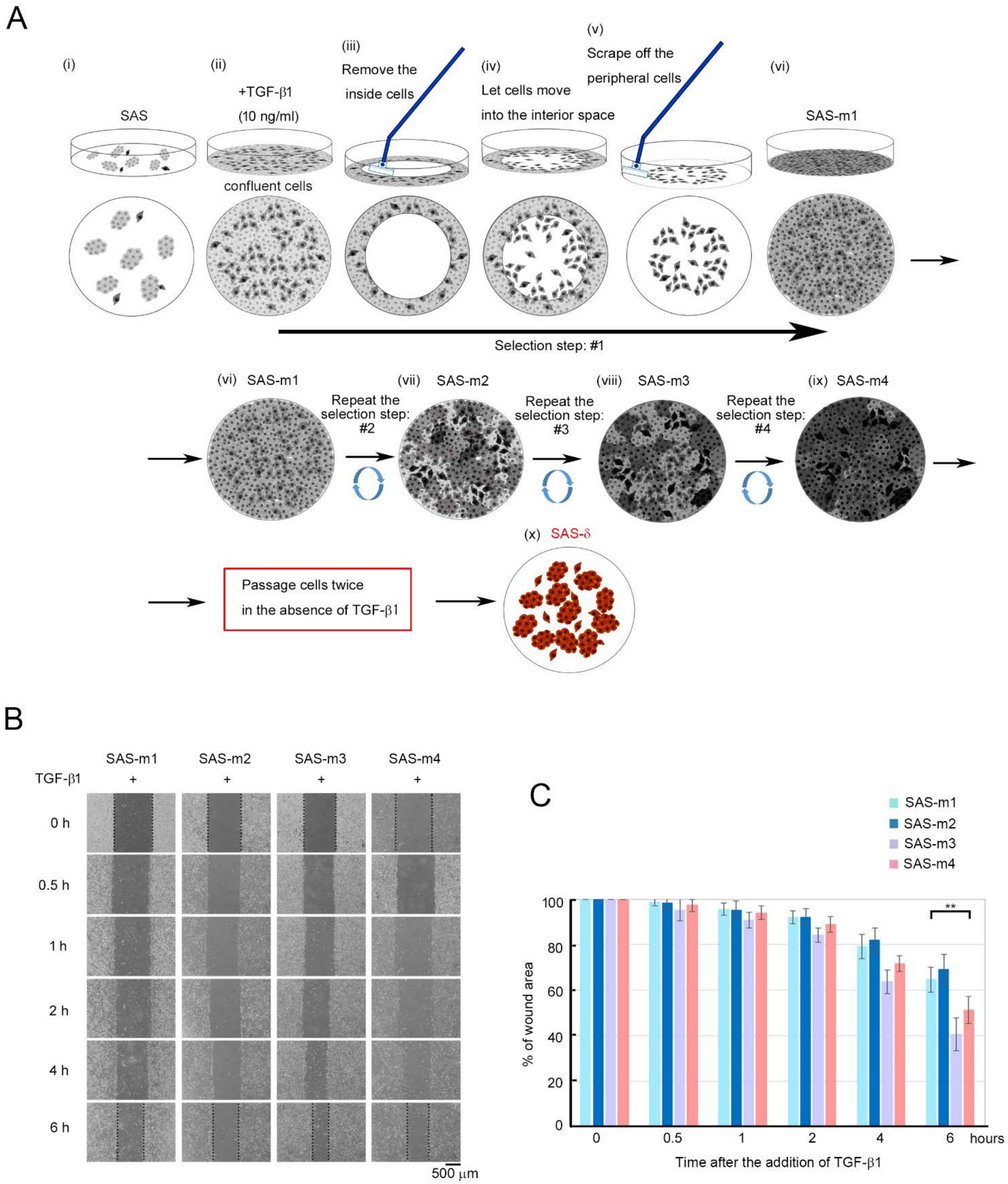

Fig. S1: Establishment of SAS-m4 and SAS-δ cell lines.

A Schematic drawings of the motility selection technique for isolation of SAS-m1, SAS-m2, SAS-m3, SAS-m4, and SAS-δ cell lines. All cell lines except for SAS-δ were cultured in DMEM + 10% FBS containing TGF-β1 (10 ng/ml) and isolated from parental SAS cells by repeating the selection step the indicated number of times (**A**-iii, removal of the central area of cells using a cell scraper; **A**-iv, allowing the outer cells to move into the interior space; **A**-v, removal of the outer cells using a cell scraper; and **A**-vi, propagation of residual cells) in the presence of TGF-β1. SAS-δ was isolated by subjecting SAS-m4 to two passages in TGF-β1-free DMEM + 10% FBS (**A**-x). See the Materials and methods section for more details. **B** Wound healing assays of the SAS-derivative cell lines in the presence of TGF-β1 (10 ng/ml). Images were acquired 0, 0.5, 1, 2, 4, and 6 h after wounding. **C** Quantification of the wound healing assays. The distances across the wound were measured at five points. The values were averaged and expressed relative to the mean value at 0 h after the addition of TGF-β1. Each value is the mean ± standard error from triplicate experiments. **, *P*<0.01.

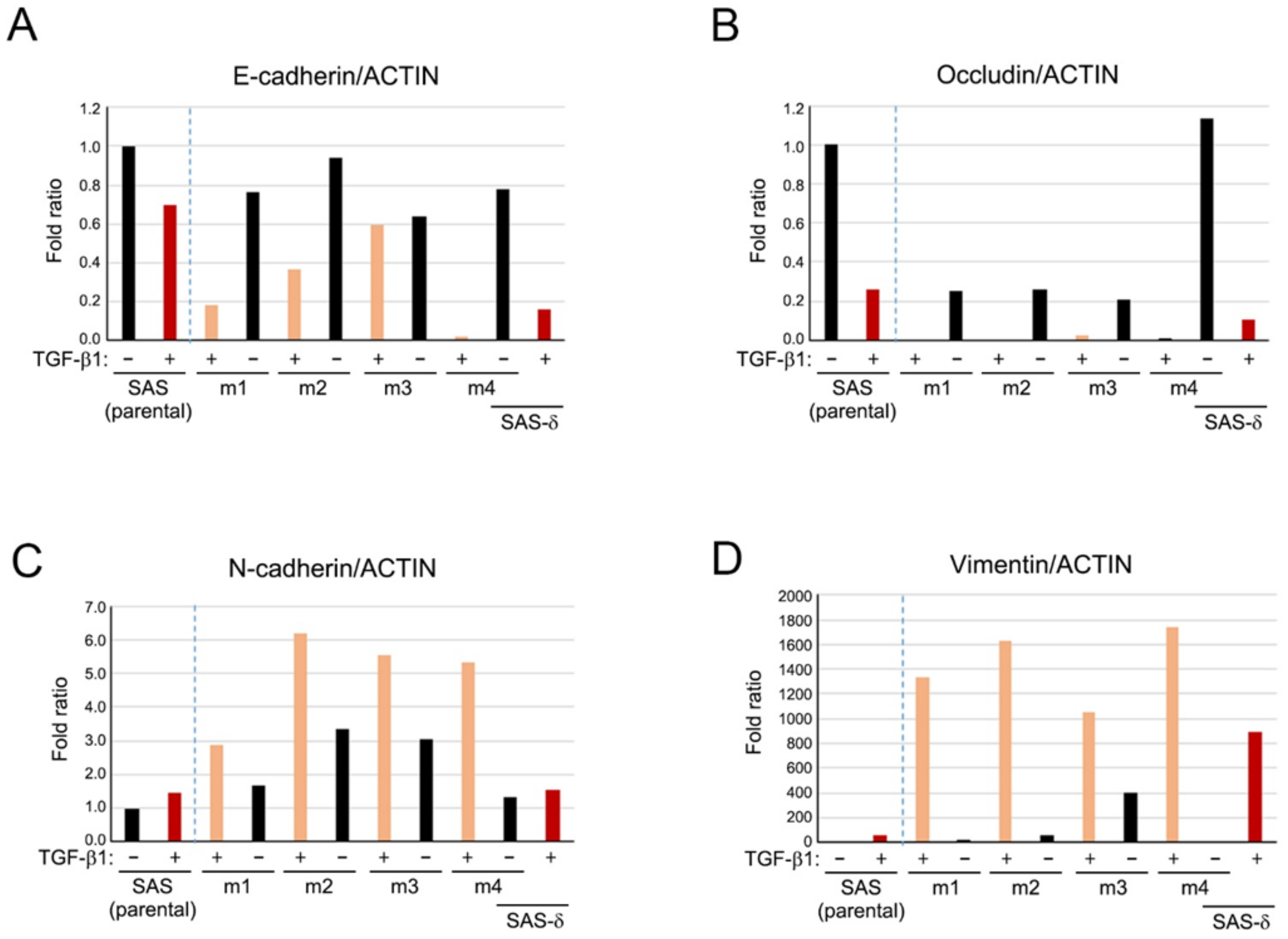

Fig. S2: Data related to Fig. 1A.

A–D Bar graphs showing the relative levels of E-cadherin (**A**), occludin (**B**), N-cadherin (**C**), and vimentin (**D**), normalized to the band intensity of ACTIN, in parental SAS and SAS derivatives with (+; red and orange bars) or without (–; black bars) TGF-β1. Blue dashed lines show the border between parental SAS and SAS derivatives (n=1). SAS-m4 cultured without TGF-β1 is equal to SAS-δ.

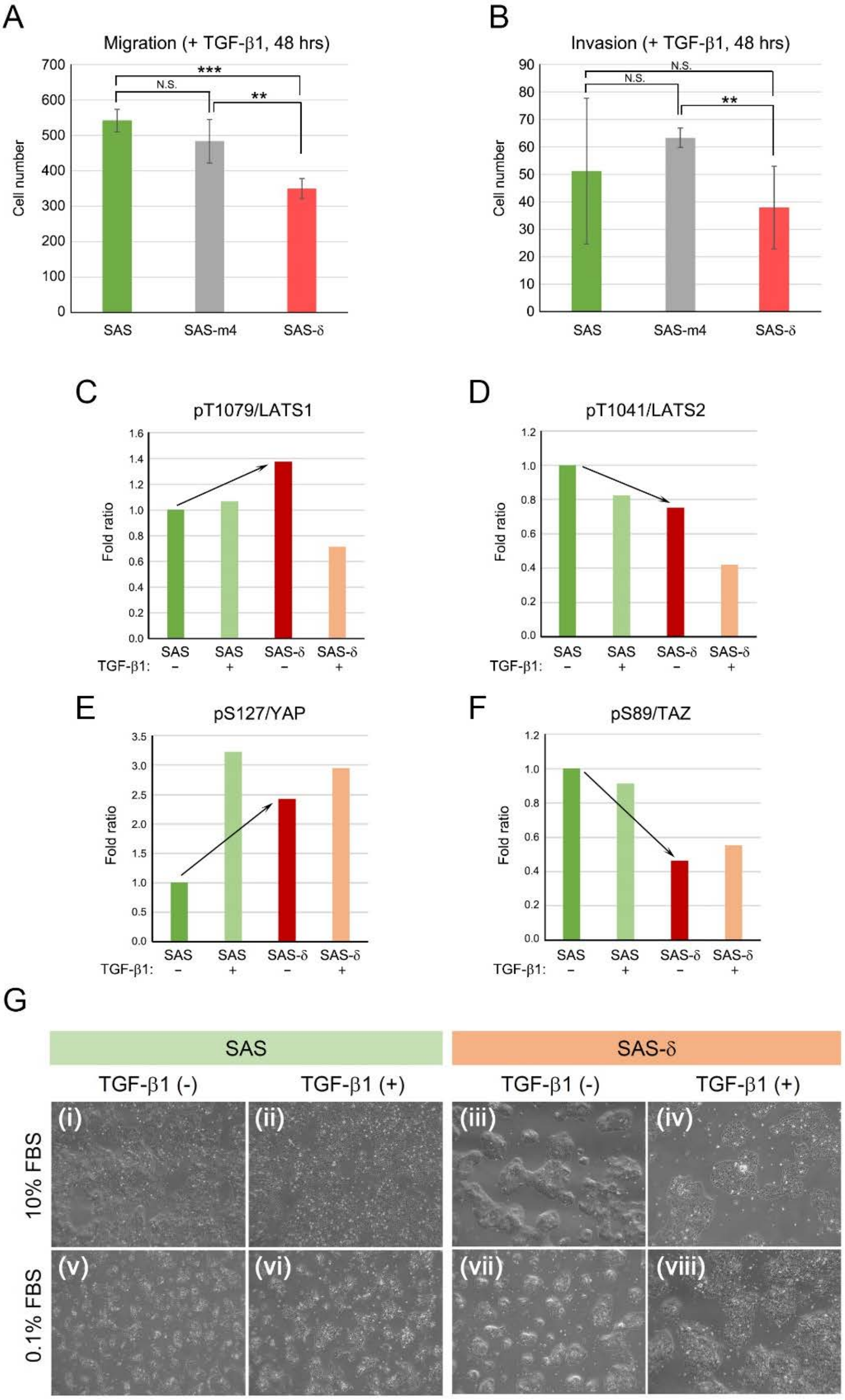

Fig. S3: Data related to Figs. 2 and 4.

A and **B** Invasion assays and migration assays of parental SAS, SAS-m4, and SAS-δ were performed in the presence of TGF-β1 (10 ng/ml, 48 h) using Matrigel BioCoat invasion chambers and the BioCoat control inserts, respectively. Cell staining was performed as in Fig. 2. Migrating (**A**) or invading (**B**) cells on the membrane were counted on images of the membrane captured through a microscope. Bar graphs show the average number of cells per microscopic field over five fields (migrating cells) or ten fields (invading cells). **, P<0.01; ***, P<0.001; N.S., not significant. **C–F** Bar graphs showing the relative levels of the indicated phosphorylated forms, normalized to the corresponding band intensity of the indicated proteins (n=1). Arrows show that the phosphorylation levels of LATS1-pT1079 and YAP-pS127 were increased in SAS-δ, whereas those of LATS2-pT1041 and TAZ-pS89 were decreased in SAS-δ under TGF-β1-free conditions. **G** Parental SAS and SAS-δ cells (5.0×10^5 cells/dish) were seeded in 100 mm dishes and grown in medium containing 10% or 0.1% FBS with (+) or without (–) TGF-β1 for 2 days. Images show the morphology of the growing cells.

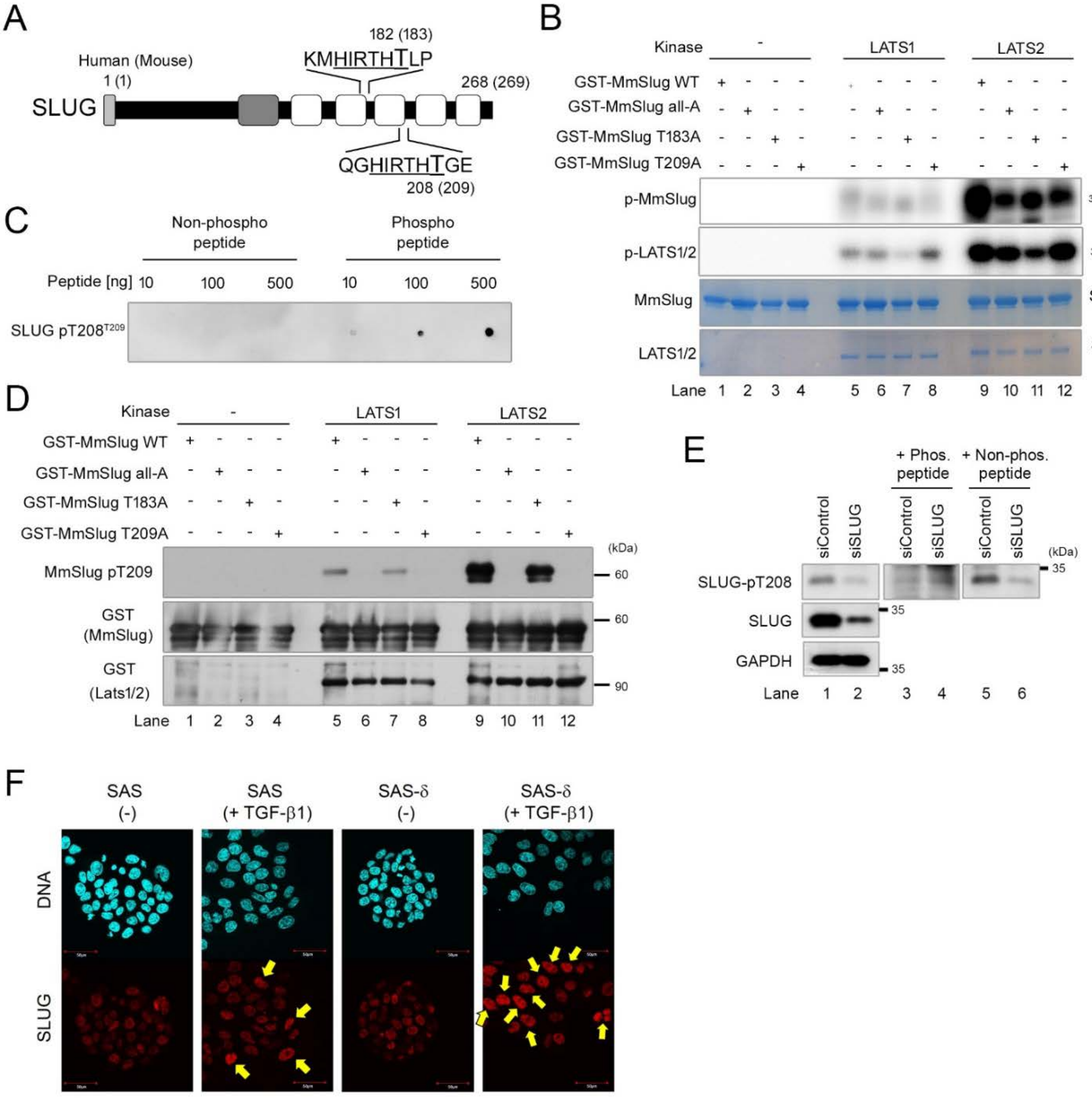

Fig. S4: SLUG-T208 is phosphorylated by LATS1/2 kinases and the nuclear localization of SLUG is enhanced by TGF-β1 treatment in SAS and SAS-δ.

A Schematic representation of SLUG protein. The light gray box, dark gray box, and five white boxes indicate the SNAG domain, SLUG domain, and zinc-finger motifs, respectively. The LATS1/2 consensus sequence and predicted phosphorylation sites (T183 and T209 in mouse; T182 and T208 in human) are underlined and in large bold letters, respectively. **B** *In vitro* LATS1/2-kinase assays with GST-fused mouse Slug-WT, -all-A, -T183A, and -T209A as substrates in the presence of [γ-^{32}P] ATP. LATS2 phosphorylated the non-phosphorylatable T183A mutant of Slug more strongly than other non-phosphorylatable mutants such as T209A and all alanine (all-A; all of seven serine and two threonine residues in the LATS consensus-like motif, R-x-x-S/T and K-x-x-S/T, were substituted by alanine) mutants, despite the lower activity of LATS2 (lane 11). SimplyBlue staining was performed to visualize the amounts of loaded proteins. **C** Dot blot assay with anti-SLUG-pT208 antibody. **D** *In vitro* LATS1/2-kinase assays in the absence of [γ-^{32}P] ATP, followed by western blotting with the indicated antibodies. Phosphorylated bands were detected with anti-SLUG-pT208 antibody. **E** SAS cells were transfected with siRNA against SLUG and cultured in the absence of TGF-β1, followed by western blotting with the indicated antibodies. For peptide competition assays, anti-SLUG-pT208 antibody was pre-incubated with phosphorylated or non-phosphorylated SLUG antigen peptides. GAPDH was used as a loading control. **F** Parental SAS and SAS-δ cells were cultured on cover slips in the presence (+) or absence (–) of TGF-β1 (10 ng/ml) for 48 h. Immunofluorescence staining of the cells with anti-SLUG-pT208 (red) antibody, followed by counterstaining with Hoechst 33258 (blue) for visualization of DNA. Yellow arrows indicate the nuclei with significantly increased signals of SLUG-pT208. Scale bars, 50 μm.

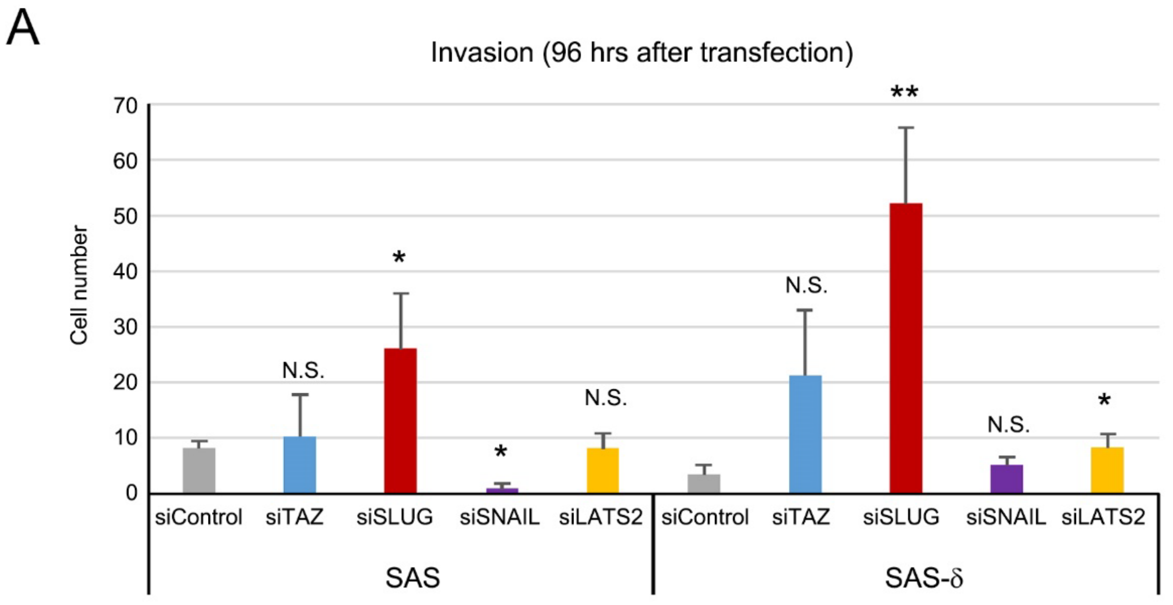

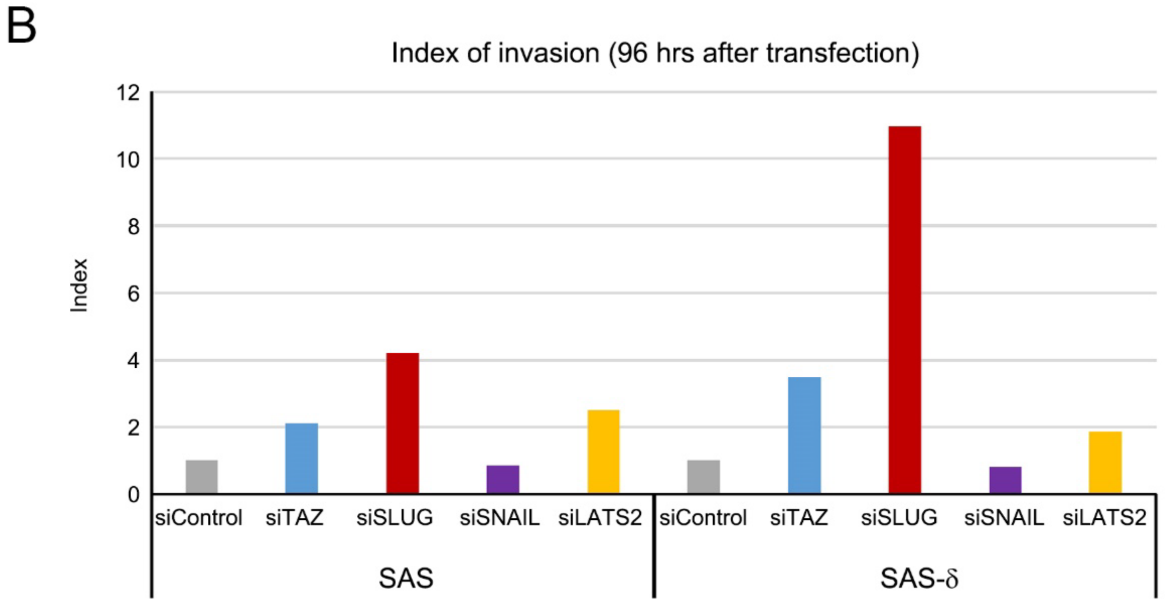

Fig. S5: Invasiveness of SAS-δ is enhanced by knockdown of SLUG but not SNAIL.
A Parental SAS or SAS-δ cells were transfected with the indicated siRNAs and invasion assays of these cells were performed in the absence of TGF-β1 using Matrigel BioCoat invasion chambers. Bar graphs show the average number of invading cells. *, $P<0.05$; **, $P<0.01$; N.S., not significant. **B** Bar graphs showing the index of the invasion shown in A, calculated by dividing the percent invasion of parental SAS or SAS-δ cells transfected with the indicated siRNAs by the percent invasion of cells transfected with siControl.

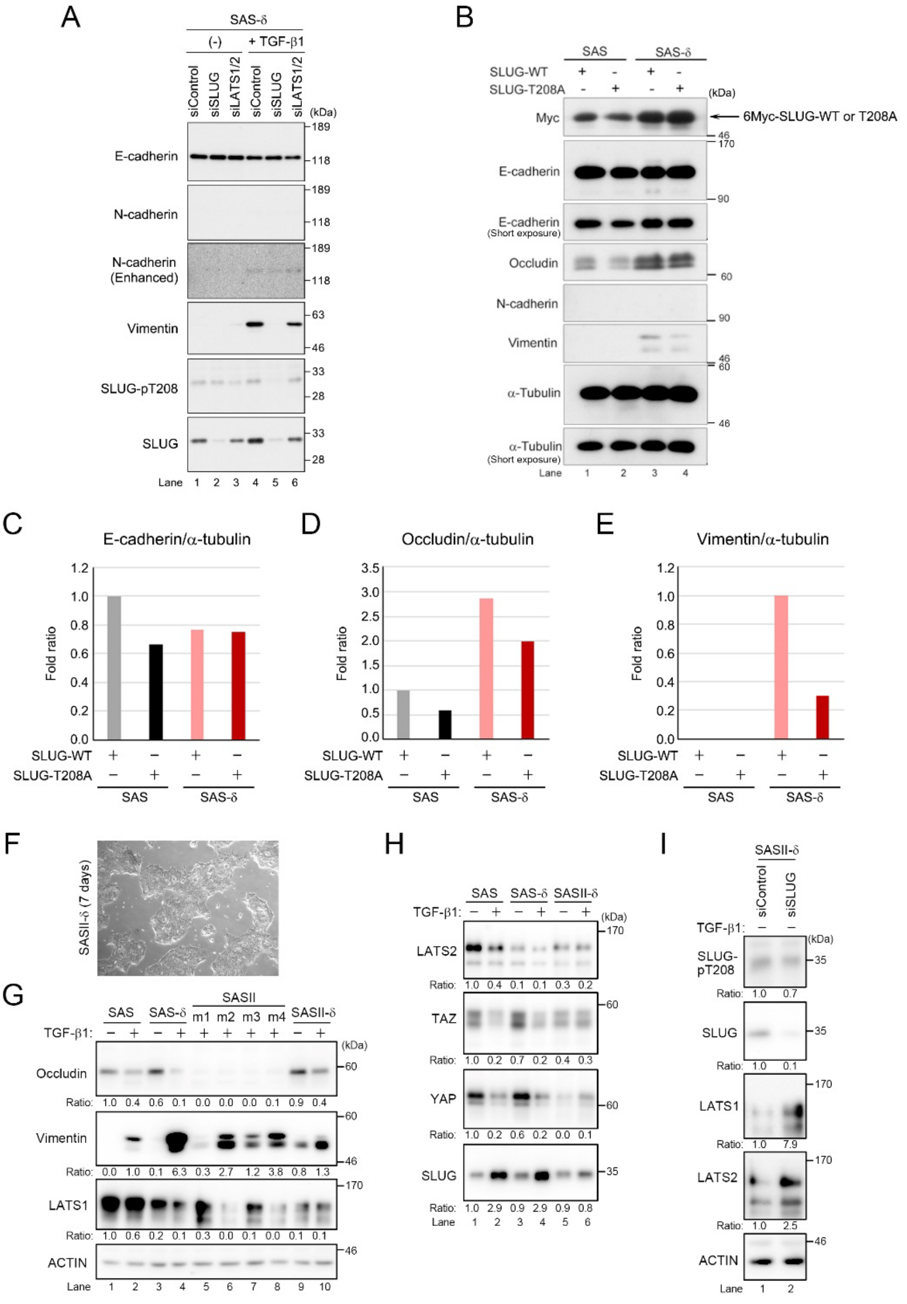

Fig. S6: Suppression of vimentin expression in SAS-δ by overexpression of the SLUG-T208A mutant and reproduction data on LATS1/2-SLUG axis using SASII-δ.

A SAS-δ cells were transfected with siSLUG, siLATS1/2 (siRNAs against LATS1 and LATS2) or siControl, and then cultured in the presence (+) or absence (–) of TGF-β1 (10 ng/ml) for 48 h as in Fig. 6D, followed by western blotting with the indicated antibodies. A loading control is shown in Fig. 6D. **B** Parental SAS and SAS-δ cells were transiently transfected with 6 x Myc-tagged SLUG-wild type (WT) or –T208A mutant, and then cultured in absence of TGF-β1 for 48 h, followed by western blotting with the indicated antibodies. α-tubulin was used as a loading control. **C–E** Relative levels of the indicated proteins in B, normalized to the corresponding band intensity of α-tubulin (n=1). **F** The photograph of SASII-δ cells on day 7 after passage in the absence of TGF-β1. **G** WB of parental SAS, SAS-δ, SASII derivatives (m1, m2, m3, and m4), and SASII-δ with the indicated antibodies against EMT-marker proteins and LATS1. ACTIN was used as a loading control. SASII-m1, -m2, -m3, and -m4 cell lines were continuously maintained in growth medium containing TGF-β1 (10 ng/ml). Parental SAS, SAS-δ, and SASII-δ were also treated with TGF-β1 for 48 h. **H** WB of parental SAS, SAS-δ, and SASII-δ with the indicated antibodies against the Hippo pathway-related proteins and SLUG. The same lysates (50 μg) as those used in G were applied in SDS-PAGE. **I** SASII-δ cells were transfected with siSLUG or siControl, and then cultured in the absence of TGF-β1 (10 ng/ml) for 48 h, followed by WB with the indicated antibodies. ACTIN was used as a loading control. **G–I** The relative levels of the indicated proteins normalized to the corresponding band intensity of ACTIN are shown below panels.

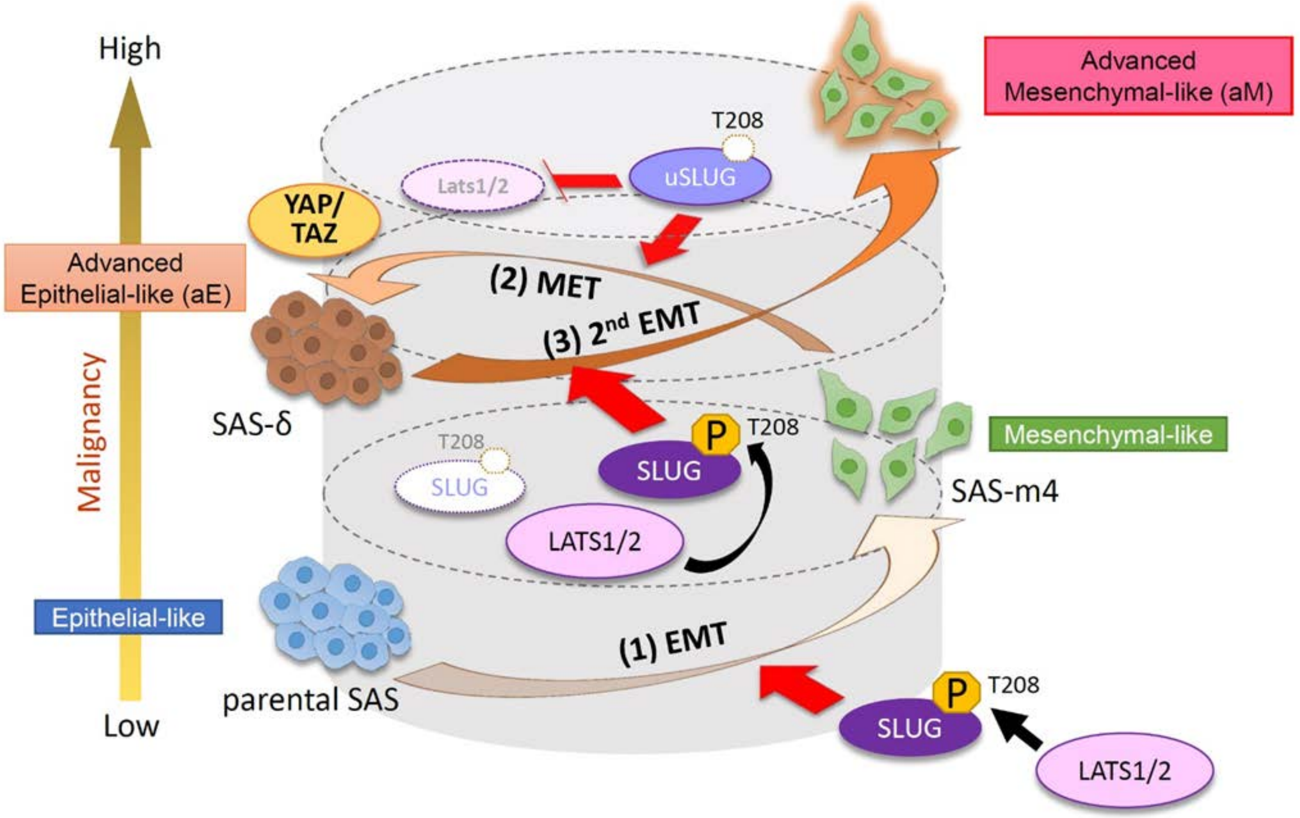

Fig. S7: A model for the evolution of cancer cells via the LATS1/2–SLUG-pT208 axis.

Schematic model of the transition from parental SAS to SAS-δ via sequential EMT/MET steps, which is associated with the canonical (LATS1/2–YAP/TAZ axis) and non-canonical (LATS1/2–SLUG-pT208 axis) Hippo pathways in the absence or presence of TGF-β1. In parental SAS, contact inhibition is likely to be induced by activation of the canonical Hippo pathway. Cancer cells are converted to a more advanced stage by progressively repeating EMT and MET as follows: (1) In parental SAS with TGF-β1, the canonical Hippo pathway is inactivated, whereas the non-canonical Hippo pathway is activated to promote EMT and cell invasion via SLUG-pT208. Epithelial-like parental SAS is transformed to mesenchymal-like SAS, such as SAS-m4. (2) Subsequently, when SAS-m4 is transformed to SAS-δ by TGF-β1 removal step, uSLUG-T208 (an T208-unphosphorylated form of SLUG) accumulates via downregulation of LATS1/2 during the transformation, which in turn, further downregulates LATS1/2 and inhibits SLUG-pT208 as a dominant-negative form in the absence of TGF-β1. In SAS-δ treated with TGF-β1, SLUG expression is transcriptionally increased by TGF-β1, and the resulting accumulation of uSLUG-T208 downregulates LATS1/2, thereby further increasing the levels of uSLUG-T208. SLUG-pT208-mediated EMT and invasion may be reversibly inhibited by the high level of uSLUG-T208 in SAS-δ. However, (3) when *de novo* expression of SLUG is accidentally suppressed by gene mutations or epigenetic dysregulation in SAS-δ (knockdown of SLUG in this study), the proportion of persistent SLUG-pT208 may increase markedly compared with uSLUG-T208, thereby inducing secondary EMT and generating advanced mesenchymal-like cancer cells in the tumor.

Fig. 1A

E-cadherin

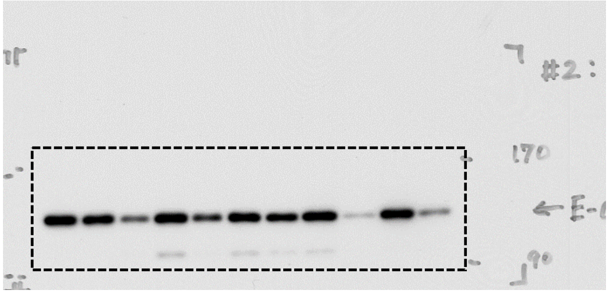

Occludin

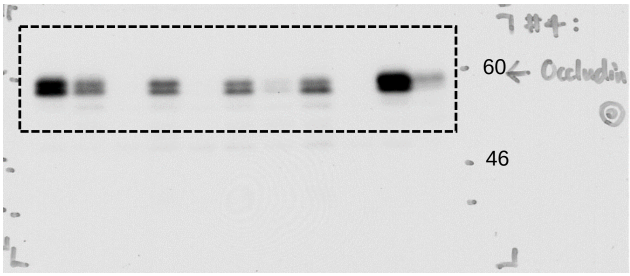

N-cadherin

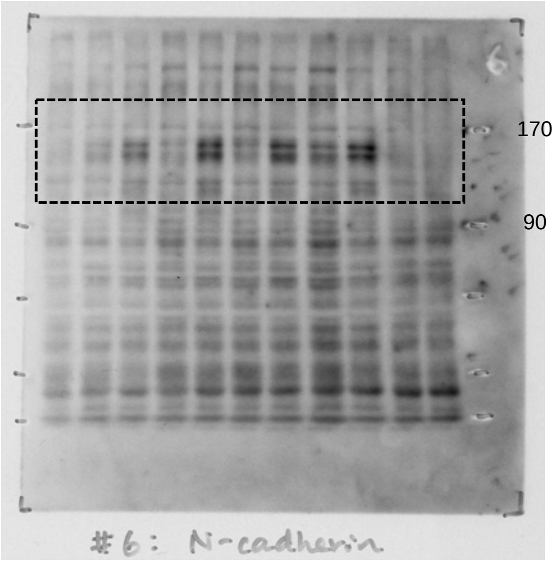

Vimentin

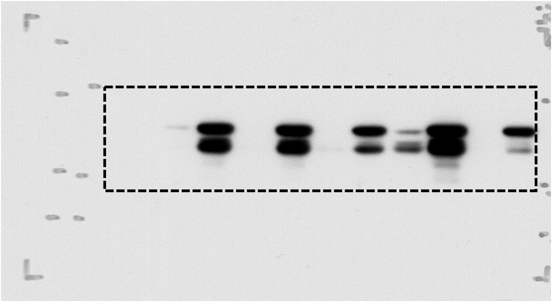

60

46

ACTIN

118
87
63
46
33
28

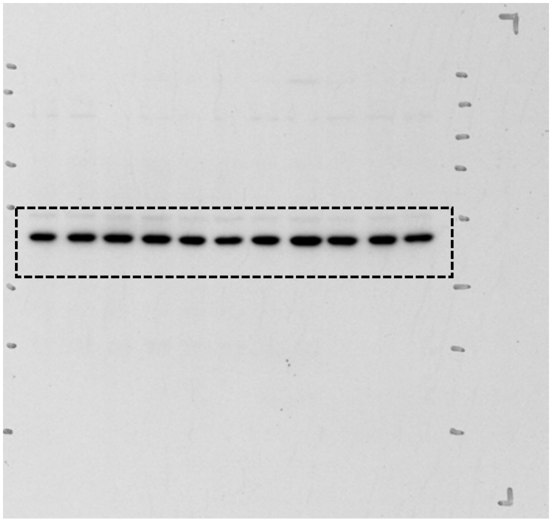

Fig. S8: Full scan images of western blots.

Fig. 4B

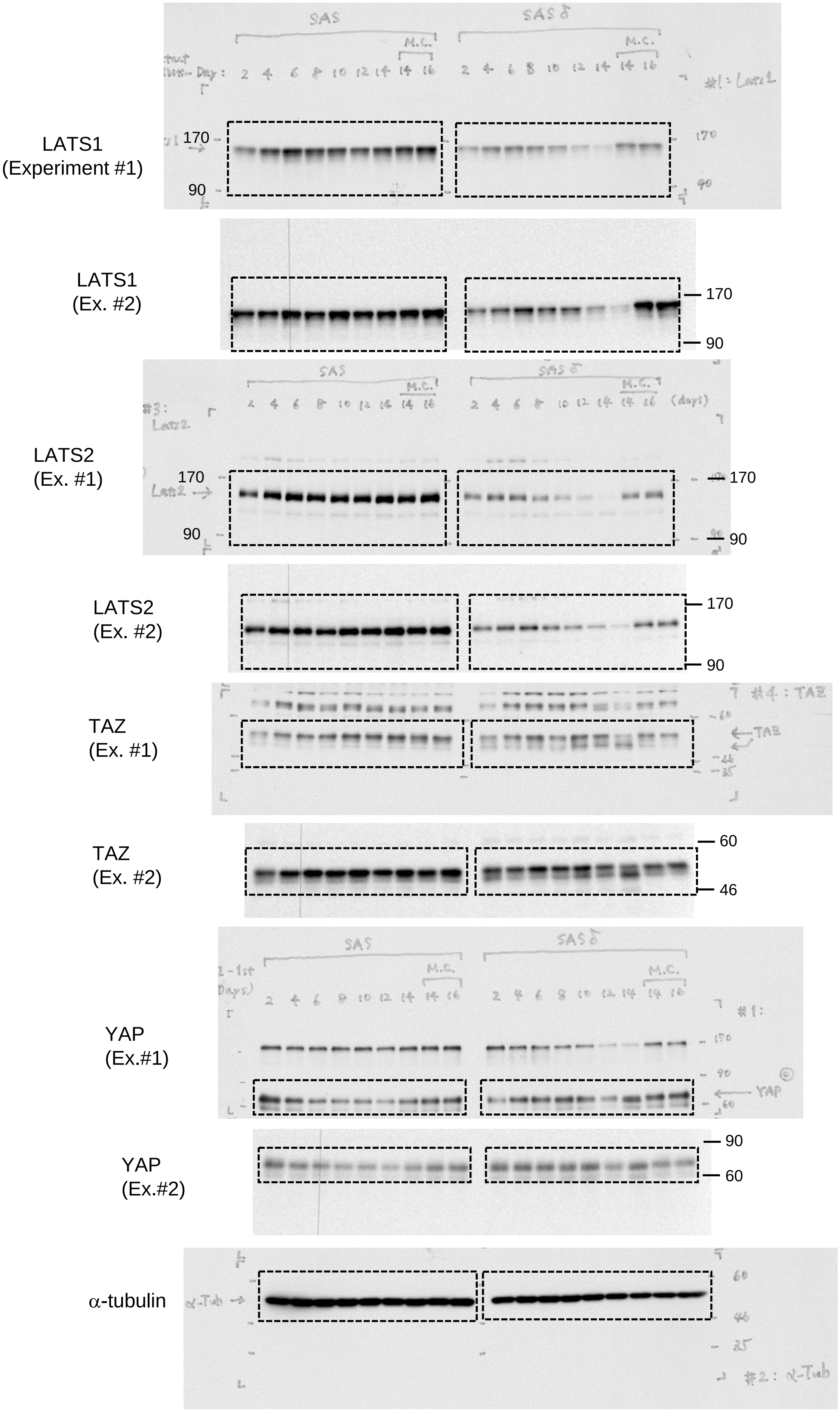

Fig. S8: Full scan images of western blots.

Fig. 4C

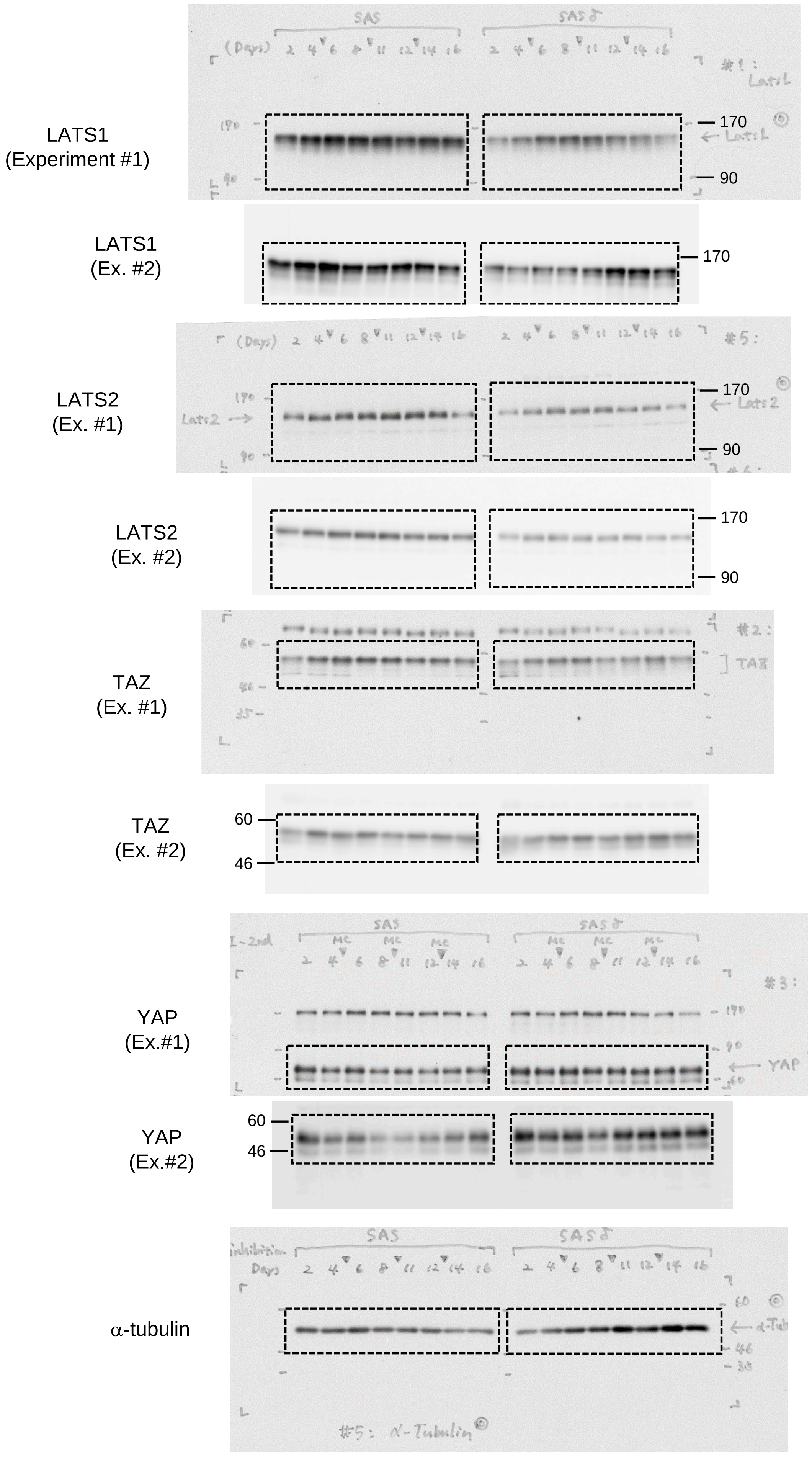

Fig. S8: Full scan images of western blots.

Fig. 4D

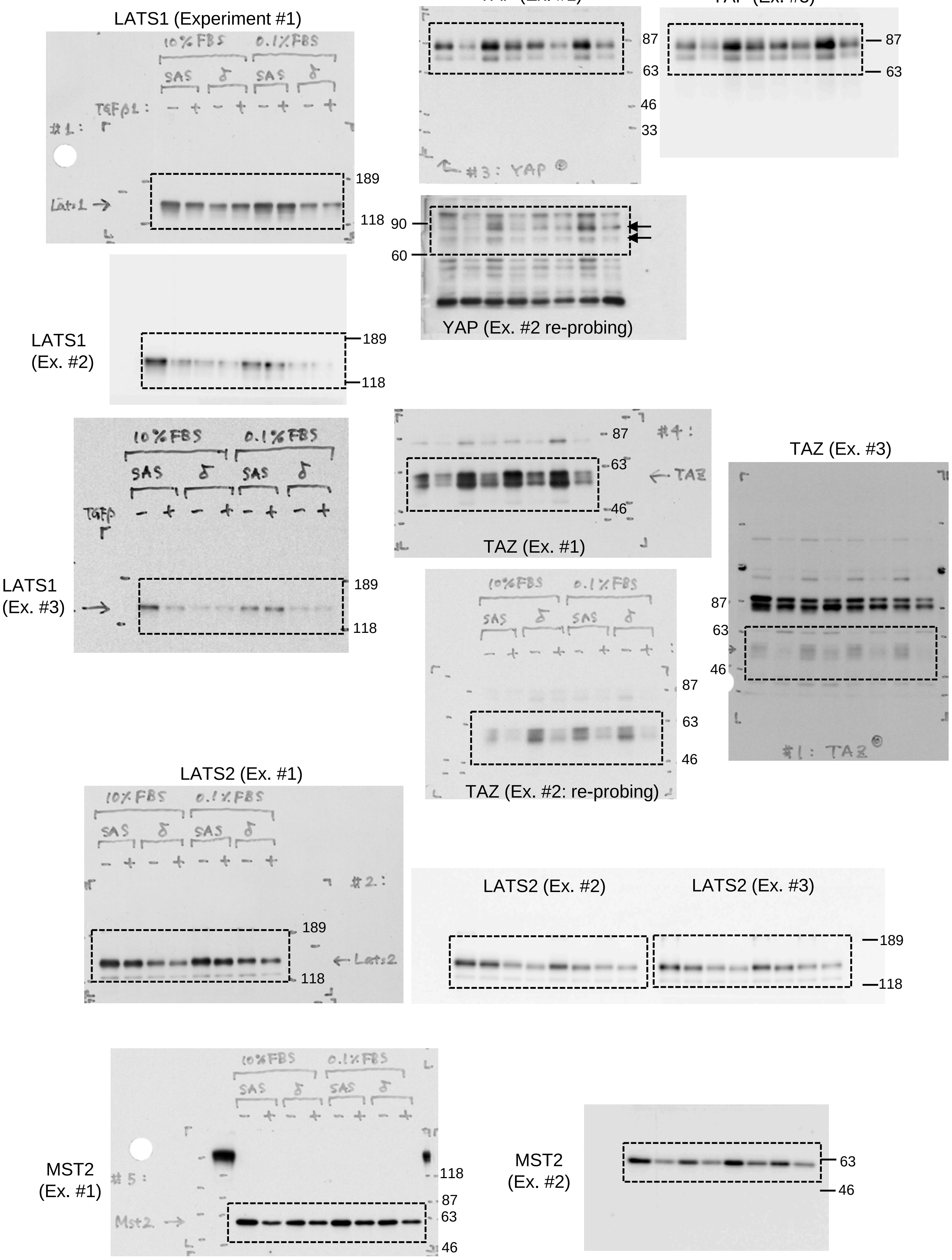

Fig. S8: Full scan images of western blots.

Fig. 4D (continued)

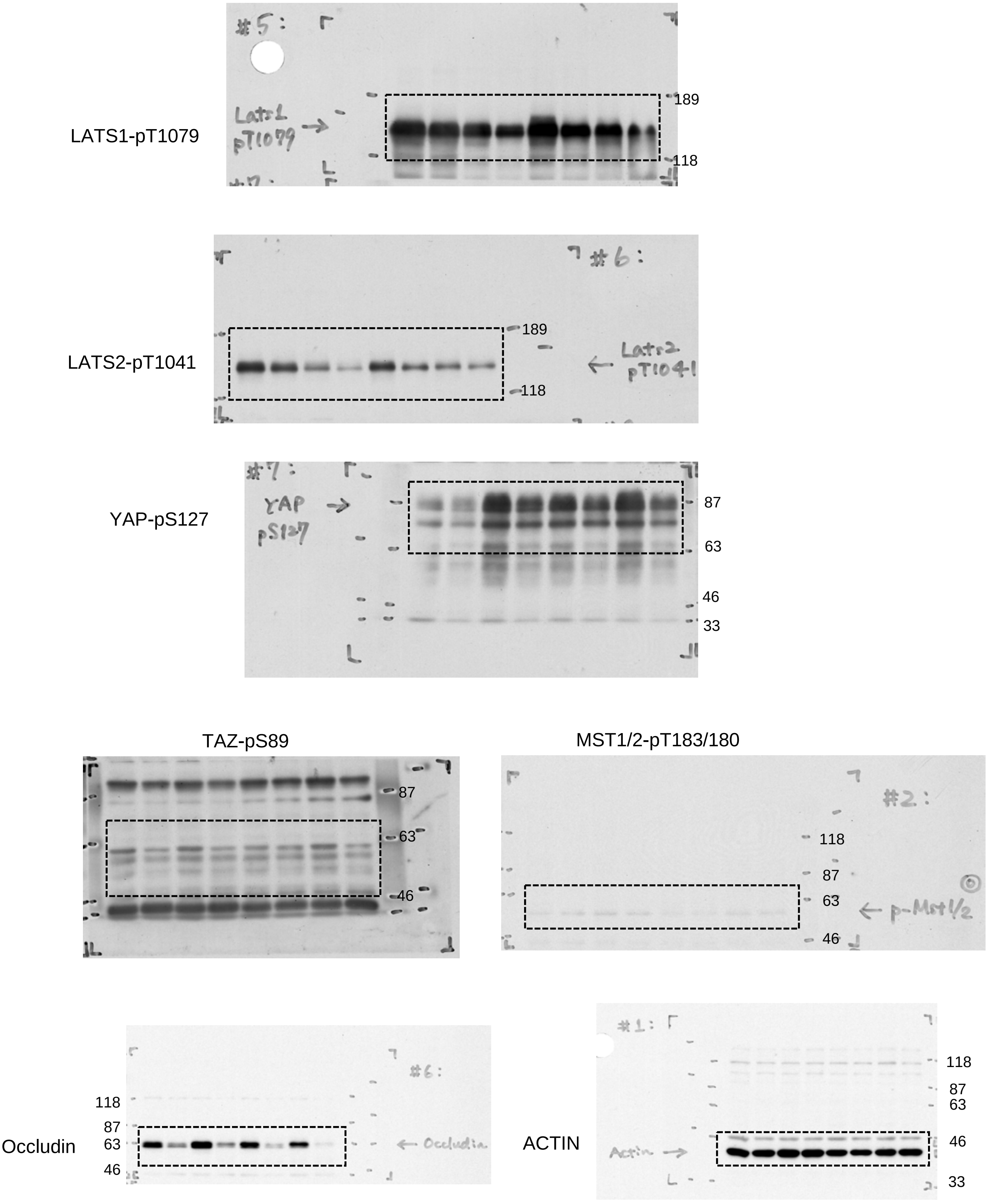

Fig. S8: Full scan images of western blots.

Fig. 5A

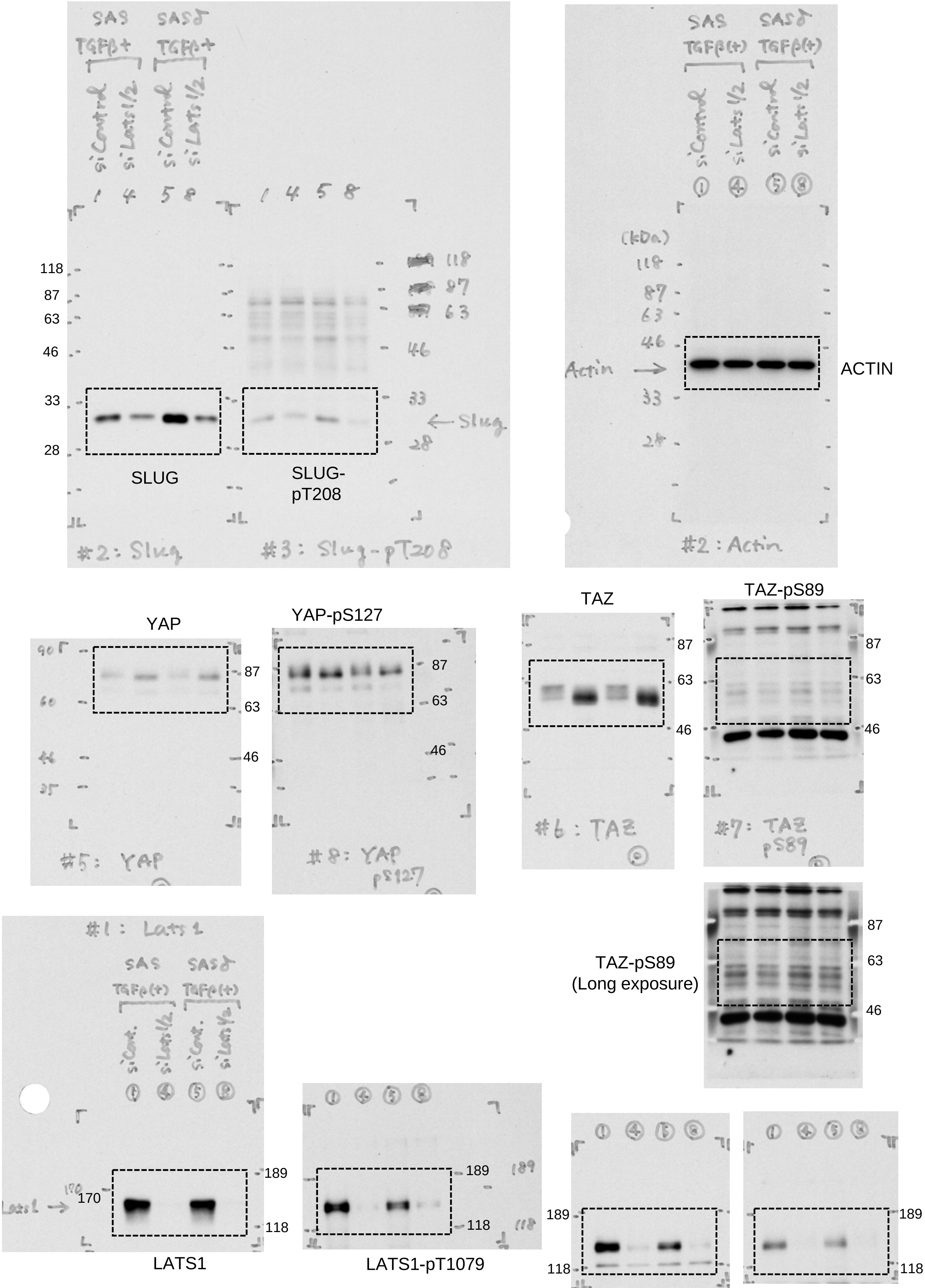

Fig. S8: Full scan images of western blots.

Fig. 5B

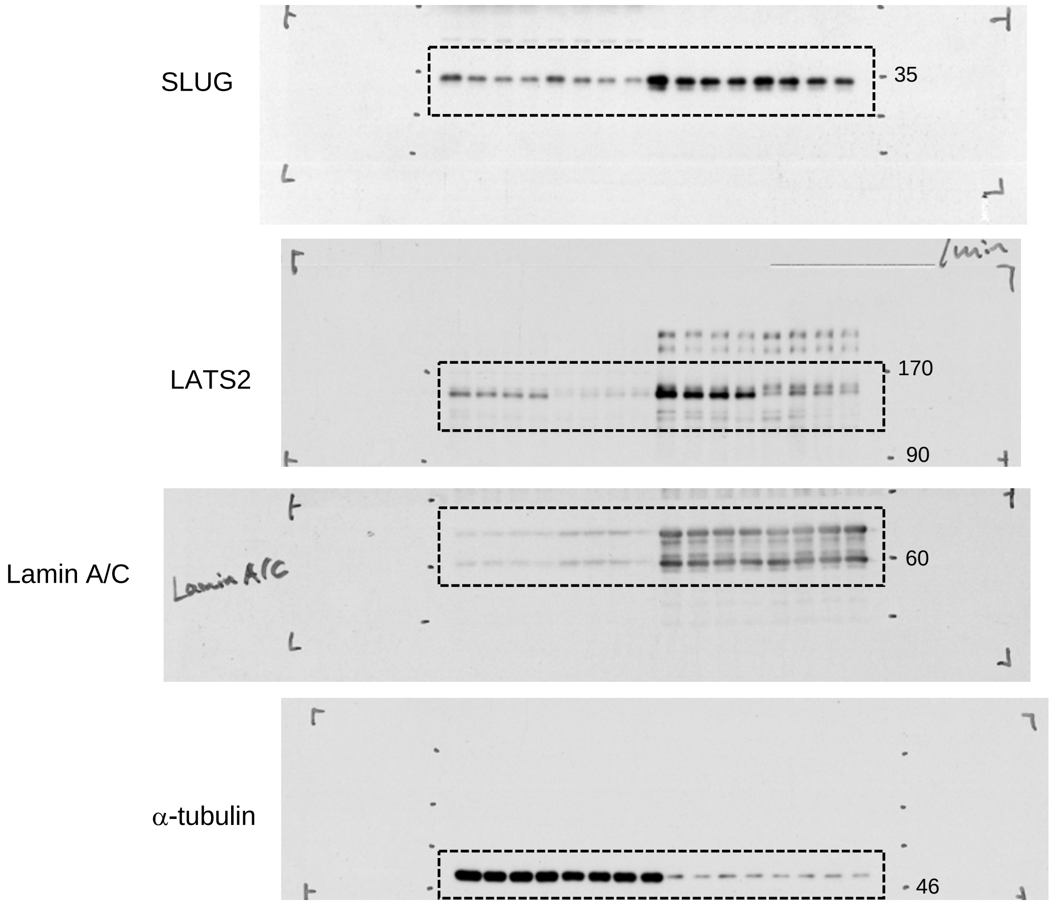

Fig. 5C

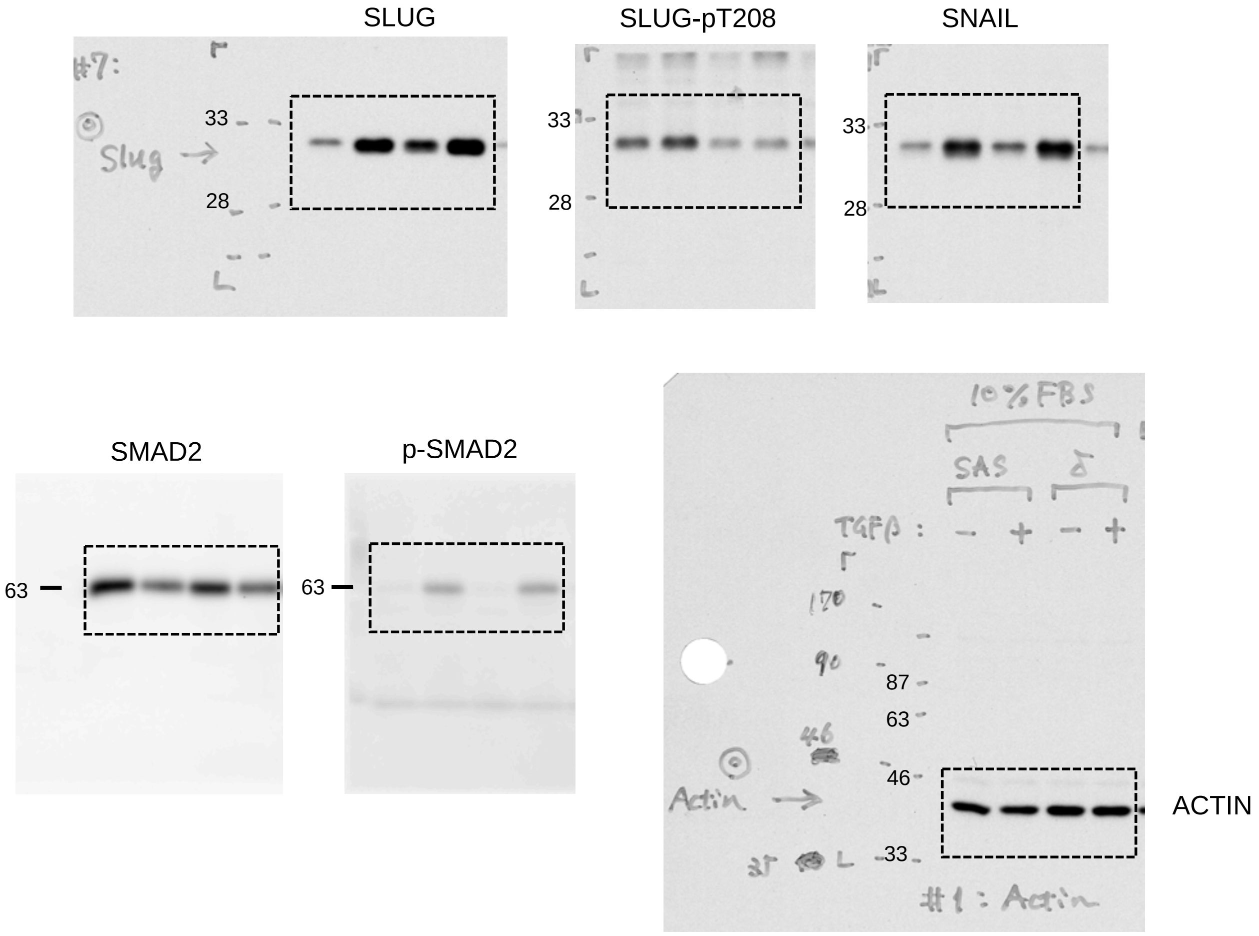

Fig. S8: Full scan images of western blots.

Fig. 6C

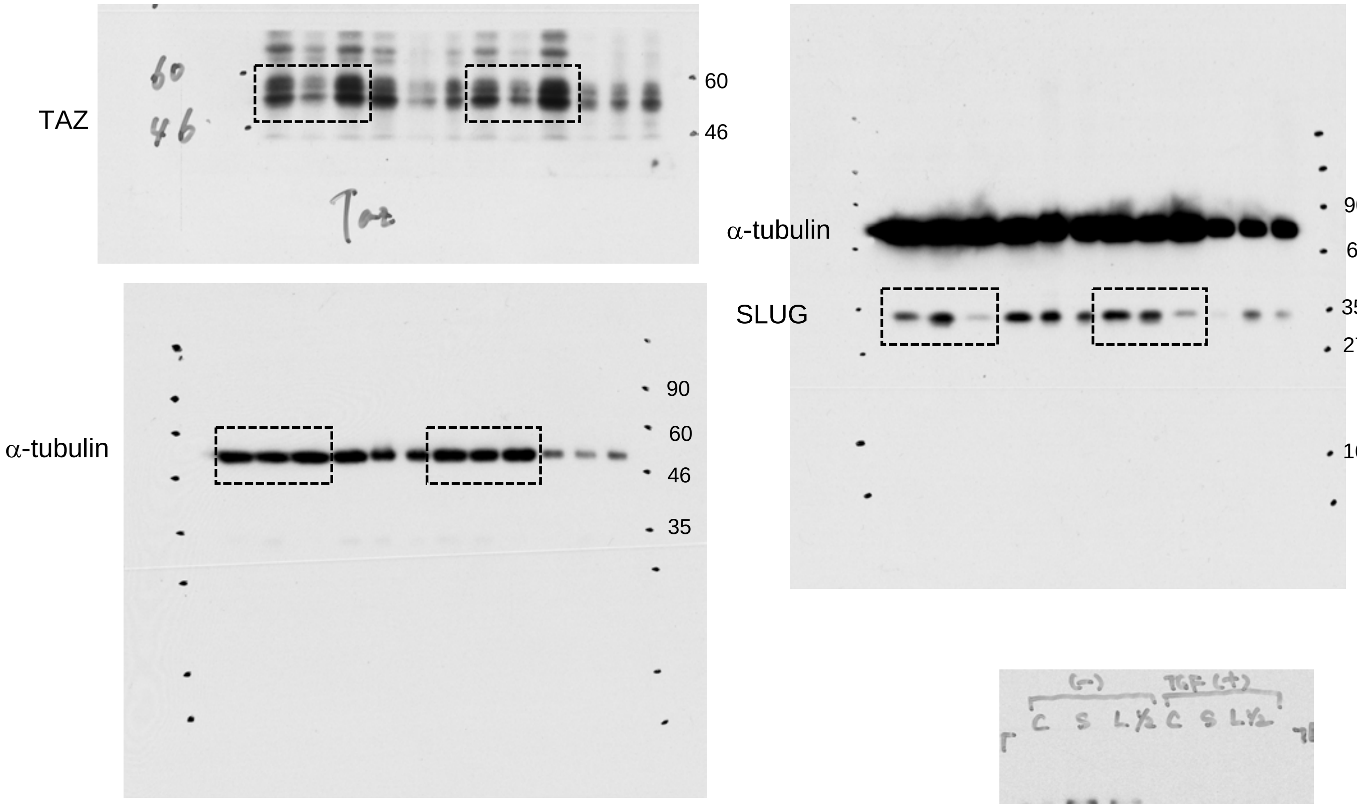

Fig. 6D

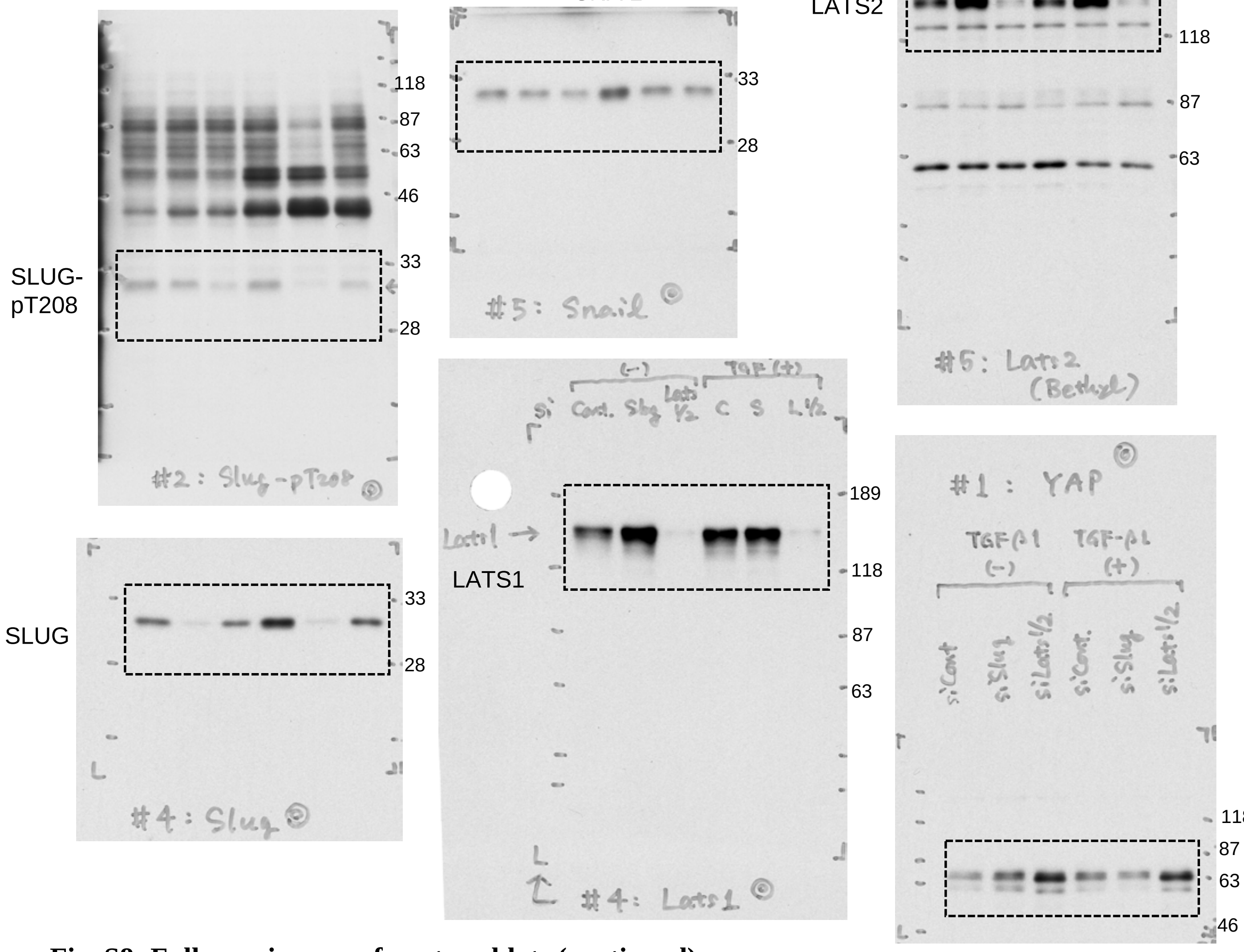

Fig. S8: Full scan images of western blots (continued).

Fig. 6C (continued)

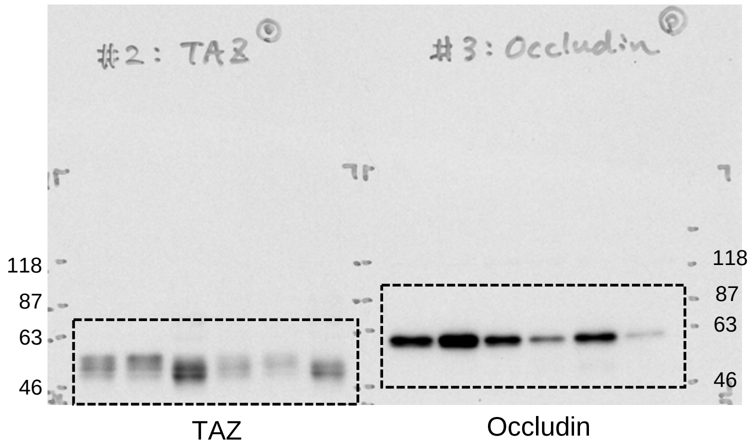

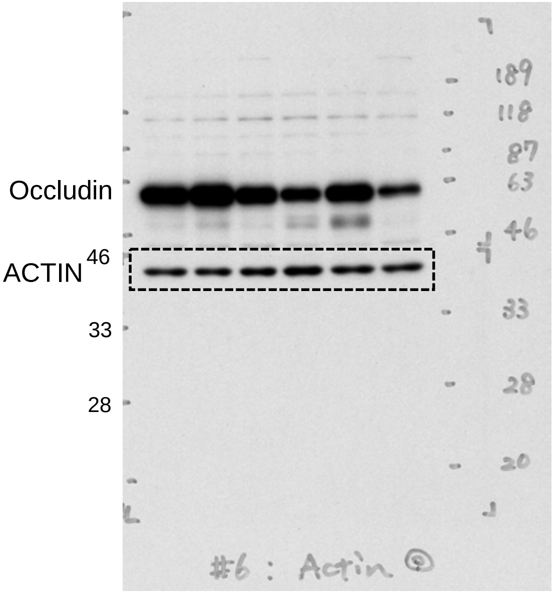

Fig. S4D

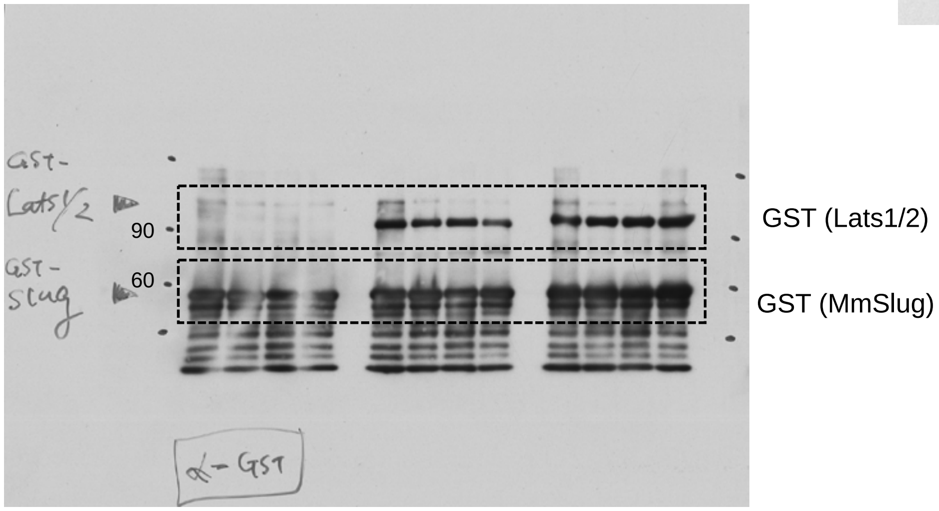

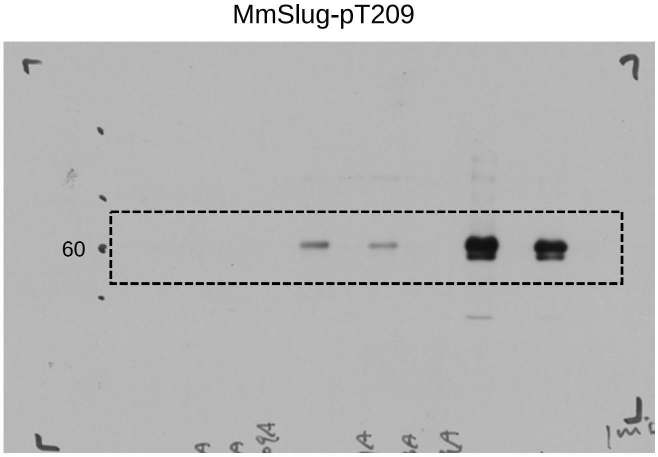

Fig. S4E

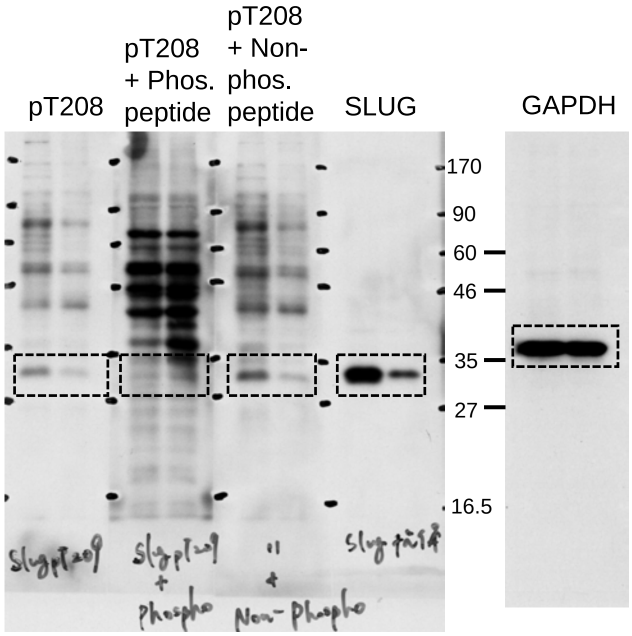

Fig. S8: Full scan images of western blots.

Fig. S6A

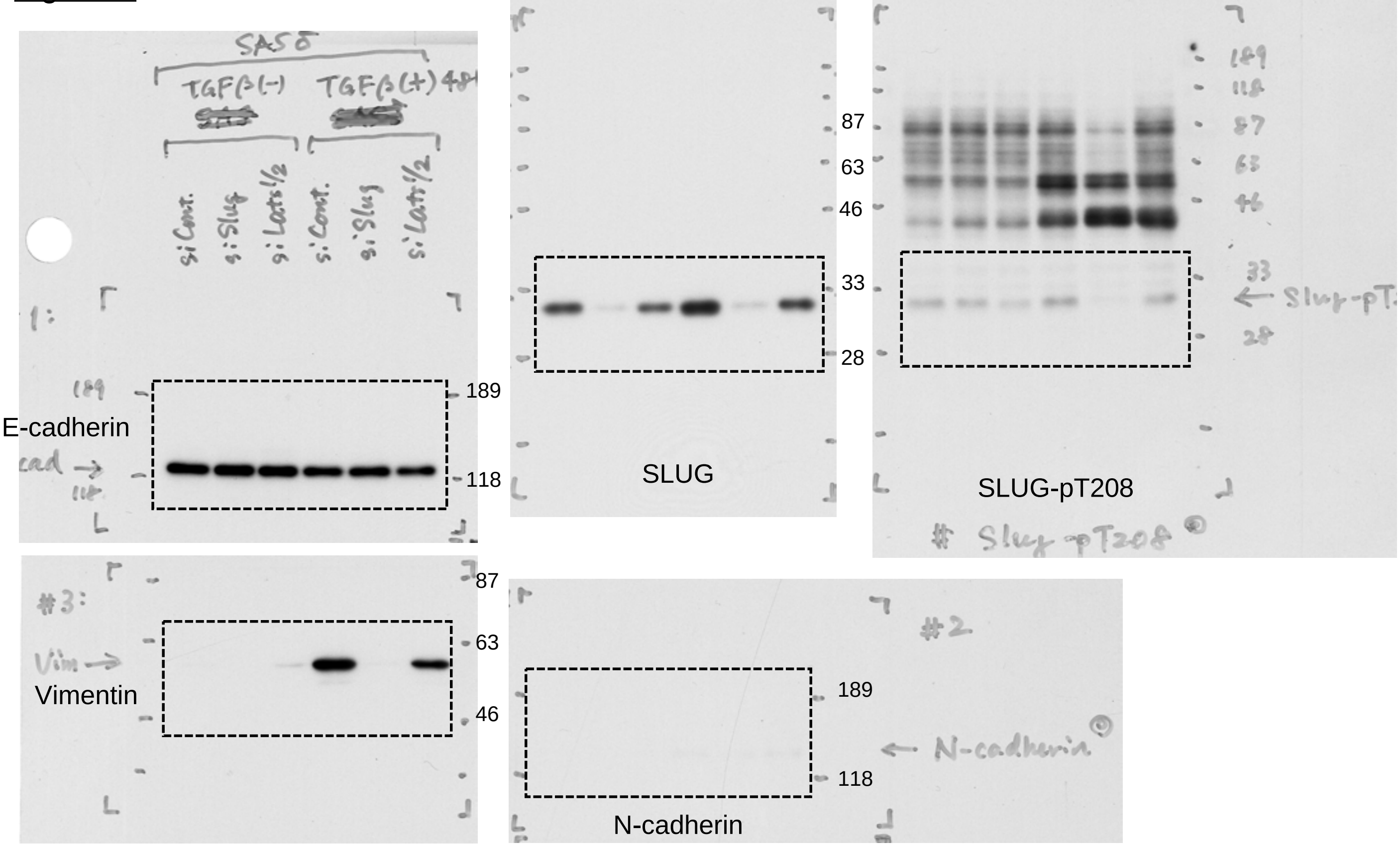

Fig. S6B

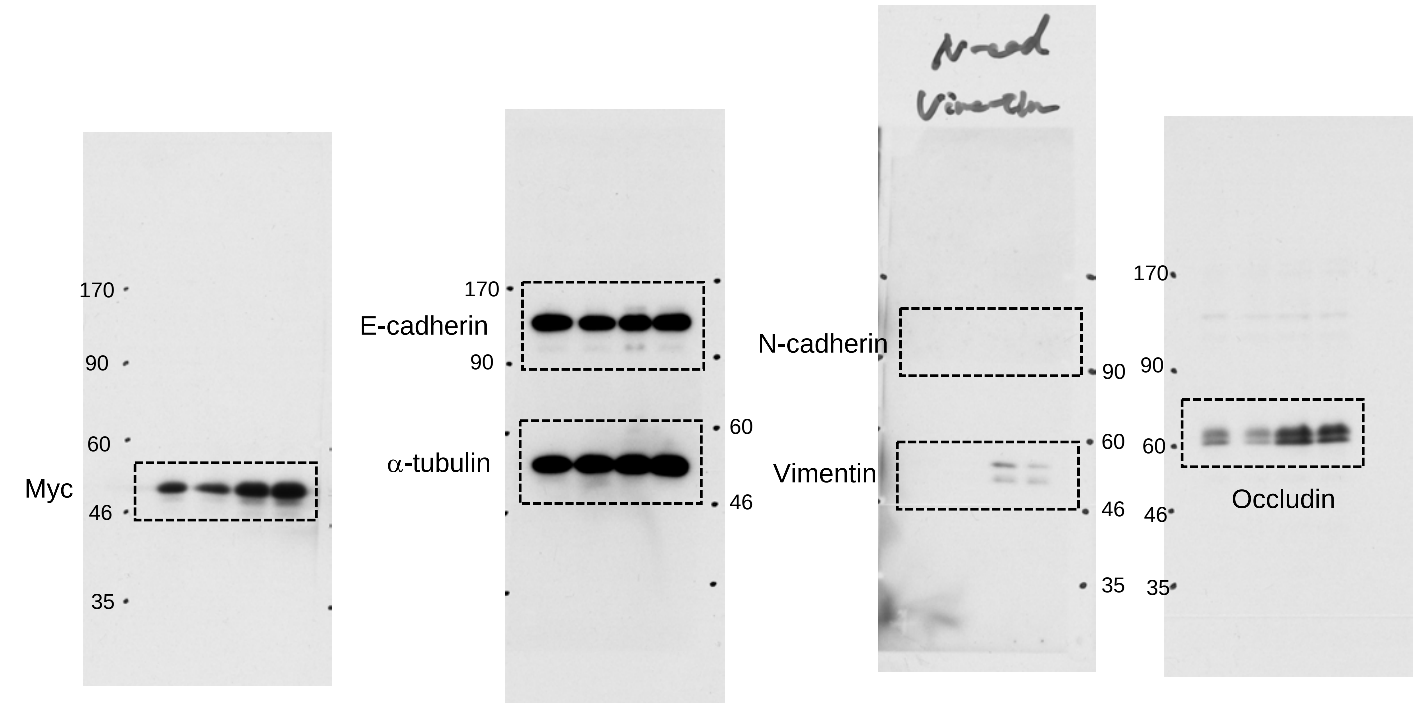

Fig. S8: Full scan images of western blots (continued).

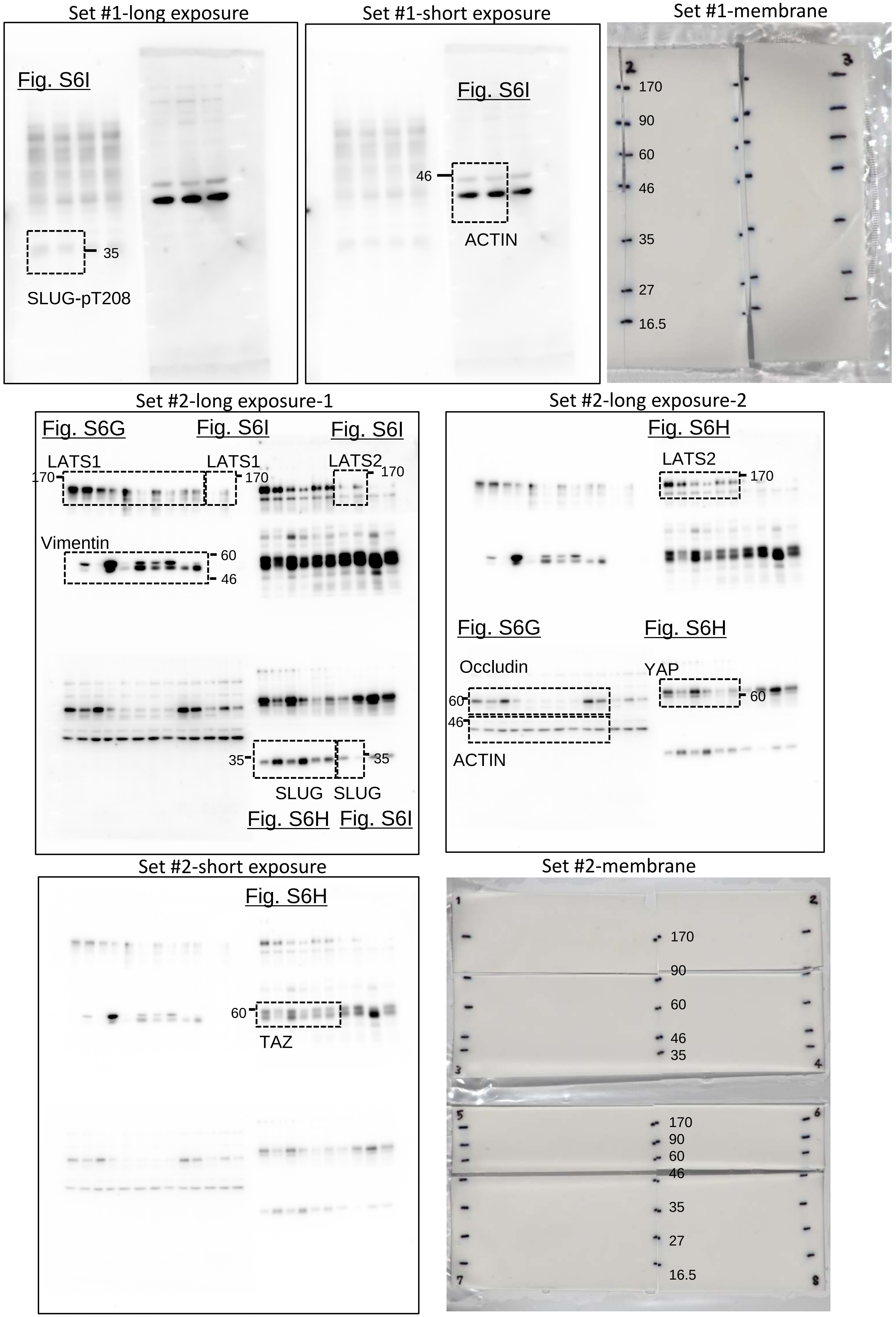

Fig. S8: Full scan images of western blots (continued).

Supplemental Table S1. Fujibayashi *et al.*

**Antibody name*<br>(*mAb, monoclonal; pAb, polyclonal)**	**Distribution source**	**Catalog No.**	**Dilution**	**Figure No.**
E-cadherin (4A2) rabbit mAb	Cell Signaling	4065	1:2000 (WB)	1A, S6A/B
Occuludin (C-terminal) rabbit pAb	SIGMA	SAB4200489	1:500 (WB)	1A, 4D, 6D, S6B/G
N-cadherin rabbit pAb	Cell Signaling	4061	1:250 (WB)	1A, S6A/B
Vimentin (D21H3) XP® rabbit mAb	Cell Signaling	5741	1:500 (WB)	1A, S6A/B/G
ACTIN (AC-40) mouse mAb	SIGMA	A4700	1:1000 (WB)	1A, 4D, 5A/C, 6D, S6G/I
LATS1 (C66B5) rabbit mAb	Cell Signaling	3477	1:500 (WB)	4B/C/D, 5A, 6D, S6G/I
LATS1-pT1079 (D53D3) rabbit mAb	Cell Signaling	8654	1:100 (WB)	4D, 5A
LATS2 rabbit pAb	BETHYL	A300-479A	1:500 (WB)	4B/C/D, 5A/B, 6D, S6H/I
LATS2-pT1041 rabbit pAb	Zhang *et al.*, 2012		1:250 (WB)	4D, 5A
YAP rabbit pAb	Cell Signaling	4912	1:500 (WB)	4B/C/D, 5A, 6D, S6H
YAP-pS127 rabbit pAb	Cell Signaling	4911	1:500 (WB)	4D, 5A
TAZ (WWTR1) rabbit pAb	SIGMA	HPA007415	1:200 (WB)	4B/C/D, 5A, 6C/D, S6H
TAZ-pS89 rabbit pAb	SANTA CRUZ	sc-17610-R	1:50 (WB)	4D, 5A
MST2 rabbit pAb	Cell Signaling	3952	1:100 (WB)	4D
MST1-pT183/MST2-pT180 rabbit pAb	Cell Signaling	3681	1:20 (WB)	4D
GST (4D8) mouse mAb	Toji *et al.*, 2004		1:1000 (WB)	S4D
SLUG (C19G7) rabbit mAb	Cell Signaling	9585	1:500 (WB)	5A/B/C, 6C/D, S4E/F, S6A/H/I
SLUG-pT208 rabbit pAb	In this study		1:100 (WB)<br>1:100 (IF)	5A/C/D, 6D, S4E, S5A, S6I
GAPDH (3H12) mouse mAb	MBL	M171-3	1:1000 (WB)	4E
SNAIL (L70G2) mouse mAb	Cell Signaling	3895	1:500 (WB)	5C, 6D
SMAD2 (D43B4) rabbit mAb	Cell Signaling	5339	1:1000 (WB)	5C
SMAD2-pS465/pS467 (138D4) rabbit mAb	Cell Signaling	3108	1:500 (WB)	5C
α-tubulin mouse mAb	SIGMA	T5168	1:2000 (WB)<br>1:150 (IF)	4B/C, 5B/D, S6B
Lamin A/C (4C11) mouse mAb	Cell Signaling	4777	1:2000 (WB)	5B
Myc (PL14) mouse mAb	MBL	M047-3	1:1000 (WB)	S6B
Anti-rabbit IgG HRP-linked antibody	Cell Signaling	7074	1:1000 (WB)	1A, 4B/C/D, 5A/B/C, 6C/D, S4C/E, S6A/B
Anti-mouse IgG HRP-linked antibody	Cell Signaling	7076	1:2000 (WB)	1A, 4B/C/D, 5A/B/C, 6C/D, S4D/E, S6B
Alexa Fluor® 488 goat anti-mouse IgG (H&L)	Invitrogen	A-11029	1:50 (IF)	5D
Alexa Fluor® 594 goat anti-rabbit IgG (H&L)	Invitrogen	A-11012	1:50 (IF)	5D, S4F
